# Circulating micronutrient levels and their association with sepsis susceptibility and severity: a Mendelian randomization study

**DOI:** 10.3389/fgene.2024.1353118

**Published:** 2024-02-16

**Authors:** Zhengxiao Wei, Yingfen Liu, Xue Mei, Jing Zhong, Fuhong Huang

**Affiliations:** ^1^ Department of Clinical Laboratory, Public Health Clinical Center of Chengdu, Chengdu, Sichuan, China; ^2^ Department of Infectious Diseases, Public Health Clinical Center of Chengdu, Chengdu, Sichuan, China; ^3^ Department of Ultrasound, Sichuan Provincial People’s Hospital, University of Electronic Science and Technology of China, Chengdu, China

**Keywords:** micronutrients, Mendelian randomization, sepsis, susceptibility, severe sepsis-related death within 28 days, zinc

## Abstract

**Background:** Sepsis, a global health challenge, necessitates a nuanced understanding of modifiable factors for effective prevention and intervention. The role of trace micronutrients in sepsis pathogenesis remains unclear, and their potential connection, especially with genetic influences, warrants exploration.

**Methods:** We employed Mendelian randomization (MR) analyses to assess the causal relationship between genetically predicted blood levels of nine micronutrients (calcium, β-carotene, iron, magnesium, phosphorus, vitamin C, vitamin B6, vitamin D, and zinc) and sepsis susceptibility, severity, and subtypes. The instrumental variables for circulating micronutrients were derived from nine published genome-wide association studies (GWAS). In the primary MR analysis, we utilized summary statistics for sepsis from two independent databases (UK Biobank and FinnGen consortium), for initial and replication analyses. Subsequently, a meta-analysis was conducted to merge the results. In secondary MR analyses, we assessed the causal effects of micronutrients on five sepsis-related outcomes (severe sepsis, sepsis-related death within 28 days, severe sepsis-related death within 28 days, streptococcal septicaemia, and puerperal sepsis), incorporating multiple sensitivity analyses and multivariable MR to address potential heterogeneity and pleiotropy.

**Results:** The study revealed a significant causal link between genetically forecasted zinc levels and reduced risk of severe sepsis-related death within 28 days (odds ratio [OR] = 0.450; 95% confidence interval [CI]: 0.263, 0.770; *p* = 3.58 × 10^−3^). Additionally, suggestive associations were found for iron (increased risk of sepsis), β-carotene (reduced risk of sepsis death) and vitamin C (decreased risk of puerperal sepsis). No significant connections were observed for other micronutrients.

**Conclusion:** Our study highlighted that zinc may emerges as a potential protective factor against severe sepsis-related death within 28 days, providing theoretical support for supplementing zinc in high-risk critically ill sepsis patients. In the future, larger-scale data are needed to validate our findings.

## Introduction

Sepsis is a critical health concern marked by an exaggerated immune response to infection, presenting a global public health challenge (Cecconi et al., 2018). It routinely precipitates multi-organ dysfunction, with high incidence and mortality rates (Evans et al., 2021), thereby imparting a substantial encumbrance upon societal and global healthcare infrastructures. Notwithstanding, the susceptibility and severity of sepsis are influenced by a multitude of factors (Rhee et al., 2017), accentuating the imperativeness of discerning modifiable factors for the prevention, timely diagnosis, and efficacious intervention in sepsis.

In recent years, although some factors potentially influencing sepsis have been identified, such as blood metabolites (Wei et al., 2023), body mass index (BMI) (Wang et al., 2023), insomnia (Thorkildsen et al., 2023), lifetime smoking (Zhu et al., 2023), the role of trace elements in the pathogenesis of sepsis remains unclear. Simultaneously, understanding the dysregulation of trace element metabolism in the pathogenesis of sepsis is not comprehensive (Guo et al., 2023). Numerous micronutrients have been reported to play a crucial role in the immune system, and their deficiency may severely impair host immunity, increasing the risk of infection (Gombart et al., 2020). Some studies emphasize vitamin C as a biological and theoretical basis for sepsis treatment (Spoelstra-de Man et al., 2018); however, a randomized controlled trial found no significant improvement in sepsis-related inflammation and vascular damage with vitamin C (Fowler et al., 2019). The disparity between these two study results may be influenced by factors such as sample size, follow-up time, and confounding variables. Due to the cost and practical difficulties, conducting sufficiently large randomized controlled trials is challenging, and there is limited research providing substantial support for the relationship between micronutrients and sepsis.

Mendelian randomization (MR) is an approach used to evaluate the relationship between risk factors and diseases in terms of causality. When there are no randomized controlled trials (RCTs) or new RCTs being conducted, MR becomes a valuable alternative approach that can provide dependable evidence on the causal connection between exposure and the risk of disease (Zuccolo and Holmes, 2017). In observational studies, MR utilizes genetic variation as an instrumental variable (IV) to successfully mitigate the influence of confounding factors that are difficult to control and reduces the likelihood of reverse causation.

In this study, we employ MR method to estimate the relationship between genetically predicted blood micronutrient levels and the risk of sepsis-related outcomes. We have selected nine micronutrients associated with infection [calcium (Ca), β-carotene, iron (Fe), magnesium (Mg), phosphorus, vitamin C, vitamin B6, vitamin D, zinc (Zn)] and assessed the infection risk of sepsis and its susceptibility and severity.

## Materials and methods

### Research design

**FIGURE 1:**
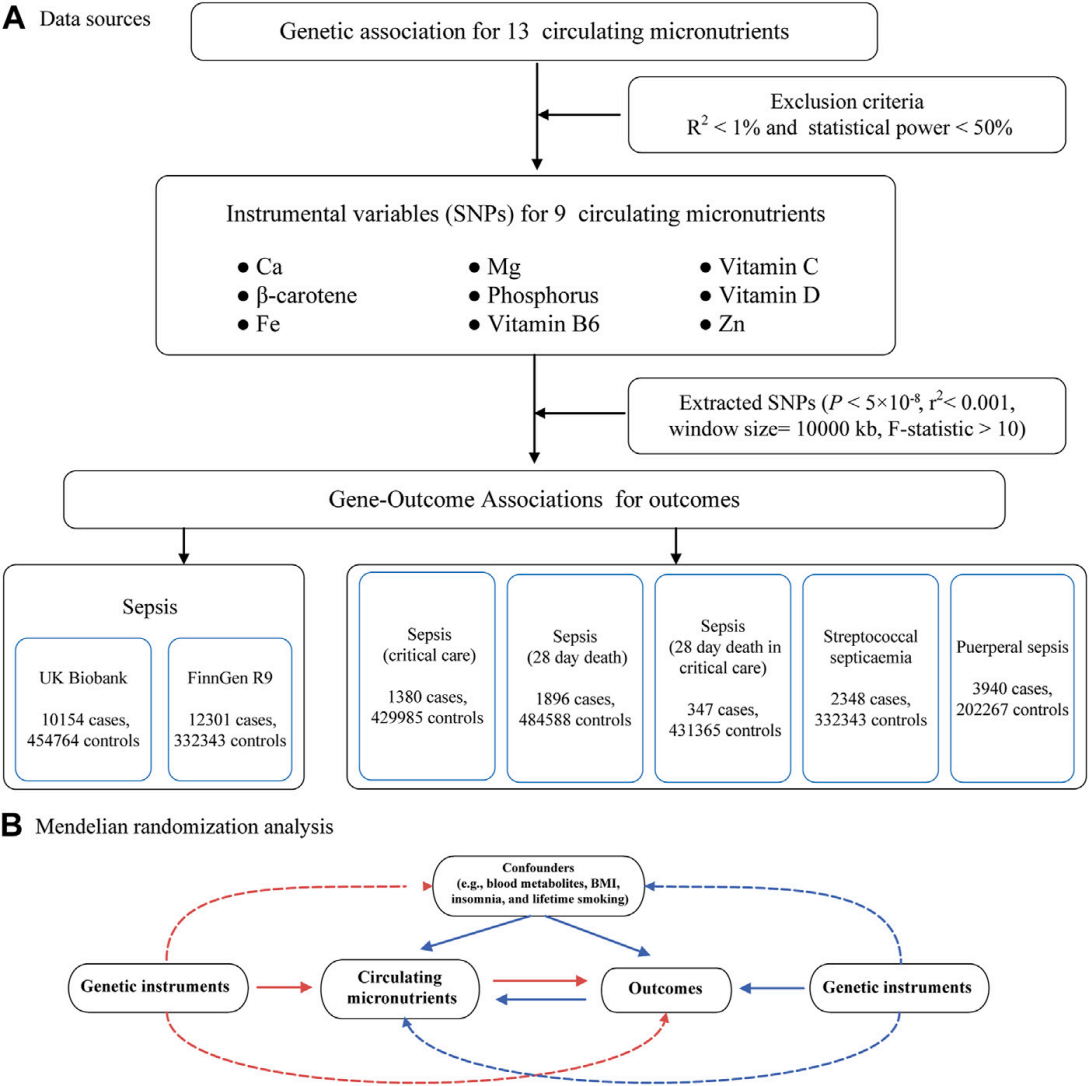
Flow chart for the Mendelian randomized analysis. **(A)** Data sources. **(B)** Mendelian randomization analysis. Abbreviations: IVW, inverse-variance weighted; Ca, Calcium; Fe, Iron; Mg, Magnesium; Zn, Zinc.

The inquiry follows the principles specified in the STROBE-MR guidelines (Supplementary Table S1). Refer to Figure 1 for an illustrative representation of the study design. In brief, we performed a comprehensive investigation of MR using data from 16 publicly accessible genome-wide association studies (GWAS) to obtain summary statistics. The objective was to elucidate the relationship between circulating micronutrient levels and sepsis. Of the 16 studies, 9 contributed exposure data, while 7 furnished outcome data. To reduce potential biases caused by population stratification, only individuals of European ancestry were included in the study for both the exposure and outcome data. For the primary MR analysis, sepsis data were procured from two independent GWAS consortia, utilized for preliminary and replicative analyses, culminating in a meta-analysis for result amalgamation. In secondary MR analyses, we scrutinized the causal nexus between micronutrients and the severity, as well as subtypes, of sepsis. Our analytic approach was bidirectional, initially scrutinizing the influence of circulating micronutrients on the susceptibility to sepsis and its associated maladies, followed by an exploration of reverse causality. A schematic overview of the study methodology is delineated in Figure 1.

The data used in this investigation were obtained from studies with the explicit consent and ethical endorsement of participants, eliminating the need for ethical approval from an institutional review board for the present study.

### Data sources for circulating micronutrient

By searching on pubmed website (https//www.ncbi.nlm.nih.gov/pubmed) (last accessed on 1 March 2023), we obtained GWAS data related to micronutrients in the European population. To prevent sample overlap between the exposures and outcomes in our research, we excluded micronutrients sourced from these two databases during our micronutrient search. No genomic studies were found for vitamin B1, B2, B3, B5, B7, fluoride, chloride, sulfur, and iodine. Exclusion of global genomic investigations on vitamin K, cobalt, chromium, sodium, potassium, and molybdenum was based on the lack of significant findings across the entire genome (Meyer et al., 2010; Dashti et al., 2014; Ng et al., 2015). A preliminary identification of 15 potential micronutrients was established: Ca (O'Seaghdha et al., 2013), copper (Evans et al., 2013), Fe (Benyamin et al., 2014), Mg (Meyer et al., 2010), selenium (Cornelis et al., 2015), Zn (Evans et al., 2013), phosphorus (Kestenbaum et al., 2010), beta-carotene (Ferrucci et al., 2009), folate (Grarup et al., 2013), vitamin A (Mondul et al., 2011), vitamin B6 (Hazra et al., 2009), vitamin B12 (Grarup et al., 2013), vitamin C (Zheng et al., 2021), vitamin D (Jiang et al., 2018), and vitamin E (Major et al., 2011). However, Vitamin A and vitamin E were not considered in these GWAS because they were controlled for body mass index (BMI), which could lead to biased genetic effects caused by BMI adjustments (Aschard et al., 2015). Detailed information about the GWAS for the 13 candidate exposures was provided in Supplementary Table S2.

### Data sources for sepsis-related outcomes

We used ICD-coded linked secondary care data to identify sepsis and sepsis-related outcomes. In the UK Biobank (Bycroft et al., 2018), sepsis and the severity were identified using ICD-10 codes A02, A39, A40, and A41. In the FinnGen database (Kurki et al., 2023), codes A40.9, A41, and O85 were used to identify sepsis and its subtypes, in line with recent literature (Hamilton et al., 2023). Cases were included if the code appeared in either the primary or secondary diagnostic position in Hospital Episode Statistics (HES) data or similar datasets in the devolved nations, as provided by the UK Biobank.

For our primary MR analysis, we chose sepsis as the primary outcome. For our study, we used summary statistics data from two separate cohorts of European ancestry, namely the UK Biobank and the FinnGen Release 9, which were employed as the outcomes. To ascertain the association of genetic variations with sepsis, we initially employed the latest version of sepsis GWAS summary data (10,154 cases and 454,764 controls) from the UK Biobank. To validate through replication and meta-analysis, we employed an additional collection of sepsis summary data (12,301 cases and 332,343 controls) obtained from the FinnGen consortium.

For secondary analyses, we opted for five sepsis-related outcomes, which encompassed three data points on sepsis severity obtained from the UK Biobank, severe sepsis (1,380 cases and 429,985 controls), and sepsis-related death within 28 days (1896 cases and 484,588 controls) and severe sepsis-related death within 28 days (347 cases and 431,365 controls). Moreover, the FinnGen cohort provided two variations of sepsis information, namely streptococcal septicaemia (2,348 cases and 332,343 controls) and puerperal sepsis (3,940 cases and 2202267 controls), which were obtained from https//r9.finngen.fi/pheno/. Refer to Table 1 for detailed information on data sources.

**TABLE 1:** Source of outcome genome-wide association study summary data.

Outcome	Source	Cases	Control	Trait/Phenocode	Population
Sepsis	UK Biobank	10,154	454,764	Sepsis	European
Sepsis	FinnGen R9	12,301	332,343	AB1_other_sepsis	European
severe sepsis	UK Biobank	1,380	429,985	Sepsis (critical care)	European
sepsis-related death within 28 days	UK Biobank	1896	484,588	Sepsis (28 day death)	European
severe sepsis-related death within 28 days	UK Biobank	347	431,365	Sepsis (28 day death in critical care)	European
Streptococcal septicaemia	FinnGen R9	2,348	332,343	AB1_strepto_sepsis	European
Puerperal sepsis	FinnGen R9	3,940	202,267	O15_puerp_sepsis	European

### MR analysis

In the selection of genomically significant SNPs, we applied stringent thresholds, specifically *p <* 5 × 10^−8^, to obtain top independent SNPs strongly correlated with each micronutrient. Within 10,000-kb windows, we eliminated single nucleotide polymorphisms (SNPs) that were in linkage disequilibrium with parameters r^2^ < 0.001. At the same time, in order to guarantee that the impact of SNPs on exposure aligns with their impact on outcomes for the identical allelic gene, we eliminated palindromic SNPs with moderate frequencies of alleles. To evaluate the statistical power, we calculated the F-statistic for every SNP. All F-statistics for the SNPs exceeded 10, indicating a minimal likelihood of weak instrumentality.

To establish the causal connection between sepsis and micronutrients, we utilized the inverse variance weighting (IVW) technique as our primary analytical method. IVW is a commonly used method in MR studies, combining the wald ratios of each SNP to derive a summary estimate (Pierce and Burgess, 2013). In order to guarantee the dependability of our findings, we performed several sensitivity analyses to confirm if diversity and pleiotropy in genetic instruments could potentially cause bias in MR outcomes. The methods used in these analyses were the weighted median, MR-Egger, MR pleiotropy residual sum, and MR-PRESSO methods. Egger intercept (Bowden et al., 2015) was utilized to evaluate horizontal pleiotropy, while the MR-PRESSO test was employed for outlier identification (Verbanck et al., 2018). Cochran’s Q test was performed to assess heterogeneity in the genetic instruments used across the two cohorts (Greco et al., 2015), with *p <* 0.05 indicating significant heterogeneity. Finally, for the outcomes of sepsis infection, an MR Steiger test was conducted to examine the directionality of the associations (Hemani et al., 2017).

To examine if the potential genetic tools related to micronutrients were linked to other traits like blood metabolites (Wei et al., 2023), BMI (Wang et al., 2023), insomnia (Thorkildsen et al., 2023), and lifetime smoking (Zhu et al., 2023), we employed PhenoScanner V2. The website http//www.phenoscanner.medschl.cam.ac.uk/ was accessed on 1 November 2023. If necessary, we assessed the correlation between exposure and outcome after excluding these SNPS from the MR analysis to mitigate potential pleiotropic effects.

Multivariable MR was employed to assess whether there was bias in any phenotype due to pleiotropy as identified on PhenoScanner. IEU OpenGWAS project (https://gwas.mrcieu.ac.uk/.) provided genetic diversity for potential pleiotropic traits. We performed a multivariable MR analysis to investigate the effect of Zn on the likelihood of severe sepsis-related death within 28 days. This analysis included mean corpuscular hemoglobin concentration (GWAS identifier: ebi-a-GCST90002328), reticulocyte count (GWAS identifier: ebi-a-GCST90025972), high light scatter reticulocyte count (GWAS identifier: ebi-a-GCST90025970), reticulocyte fraction of red cells (GWAS identifier: ebi-a-GCST90002406), and mean corpuscular volume (GWAS identifier: ebi-a-GCST90025963).

### Power statistics

We conducted power calculations using the online platform (https://shiny.cnsgenomics.com/mRnd/) (Brion et al., 2013). Based on the sample sizes used in the meta-analysis, we computed the statistical power for each analysis under a type I error of 5%, and the results are summarized in Table 2. In order to guarantee the strength of our conclusions, we exclusively took into account micronutrients that had an R^2^ value higher than 1% and/or a statistical power exceeding 50% for at least one sepsis-related outcome (Flatby et al., 2023). This criterion led to the exclusion of copper, folate, selenium, and vitamin B12 from further analysis, as detailed in Supplementary Tables S3, S4.

**TABLE 2:** Source of exposure genome-wide association study summary data.

Exposure	Number of SNPs	% Of variance explained	Population ancestry	Pubmed ID
Ca	7	0.841	European	24,068,962
Cu	2	4.6	European	23,720,494
Fe	3	3.04	European	25,352,340
Mg	5	1.49	European	20,700,443
P	5	1.2	European	20,558,539
Se	7	3.65	European	25,343,990
Folate	2	0.41	European	23,754,956
β-carotene	4	8.36	European	19,185,284
Vitamin B6	2	3.07	European	19,744,961
Vitamin B12	10	4.78	European	23,754,956
Vitamin C	11	1.79	European	33,203,707
Vitamin D	6	2.67	European	29,343,764
Zn	2	4.59	European	23,720,494

Abbreviations: Ca, Calcium; Cu, Copper; Fe, Iron; Mg, Magnesium; P, phosphorus; Se, Selenium; Zn, Zinc.

### Replication and reverse MR analysis

To perform the primary MR analysis, we carried out a replication analysis by utilizing supplementary sepsis summary data obtained from the FinnGen consortium. The findings from the two groups (UK Biobank and FinnGen) were combined and analyzed using a f random-effects model in METAL (version 2011-03-25) (Willer et al., 2010). Additionally, to further assess whether our MR study was affected by reverse causation, we performed a reverse MR analysis on the association between genetically predicted sepsis and candidate micronutrients. In this reverse MR analysis, susceptibility and severity of sepsis were treated as exposures, and candidate micronutrients were considered as outcomes. We applied the identical rigorous standards for selecting instrumental variables, requiring a significance level of *p* < 5 × 10^−8^, and ensuring linkage disequilibrium with r^2^ < 0.001 within windows of 10,000-kb.

### Statistical analysis

The TwoSampleMR package (version 0.5.6) and the R package “MRPRESSO” (version 4.0.3) were utilized for conducting all MR analyses. METAL (version 2011-03-25) (Willer et al., 2010) was utilized for meta-analysis of results. A significance threshold of *p* < 0.05 was deemed to be of nominal importance, whereas the Bonferroni-adjusted statistical significance threshold (for 9 exposures) was established at *p* = 0.05/9 = 5.56 × 10^−3^.

## Results

### Instrumental variable selection

The number of instrumental variables for circulating micronutrients ranged from 2 to 11. The F-statistics for these SNPs ranged from 25 to 1,497, with a median of 50, surpassing the conventional threshold of 10, suggesting a minimal likelihood of weak instrumentality (see Supplementary Table S4). However, it is noteworthy that one SNP for calcium (rs1550532), two SNPs for magnesium (rs7965584, rs7197653), and one SNP for vitamin D (rs8018720) were unavailable in the outcome datasets for streptococcal septicaemia and puerperal sepsis. Furthermore, the outcome dataset for severe sepsis-related death within 28 days did not include one SNP related to Fe (rs1525892), and two SNPs associated with phosphorus (rs1697421, rs9469578) were excluded due to incompatible alleles.

### Primary MR analyses

**FIGURE 2:**
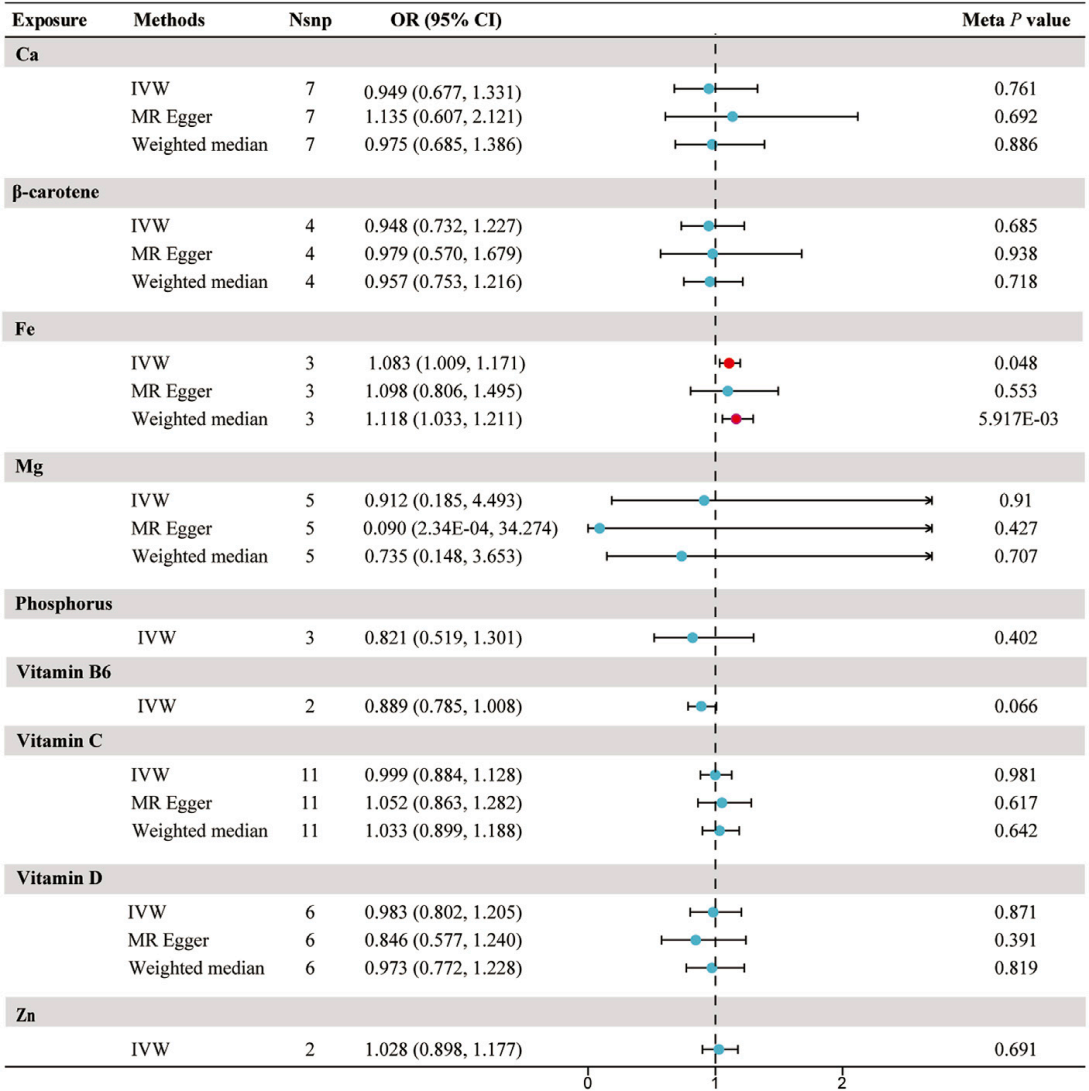
Forest plot for the meta-analysis of circulating micronutrients levels on the risk of sepsis. Abbreviations: IVW, inverse-variance weighted; Nsnp, number of SNP; OR, odds ratio; CI, confidence interval; Ca, Calcium; Fe, iron, Mg, Magnesium; Zn, zinc.

Using the chosen instrumental variables, we performed an initial evaluation on the association between 9 circulating micronutrients and sepsis likelihood in the UK Biobank discovery set. We identified two independent associations with sepsis for β-carotene and Fe (*p* < 5.56 × 10^−3^) (Supplementary Table S5). No heterogeneity was found in the sensitivity analyses, which included Cochran’s Q test and I^2^ values (β-carotene *p* = 0.997, Fe *p* = 0.264). Evaluation of horizontal pleiotropy using MR-Egger suggested insufficient evidence for horizontal pleiotropy (Supplementary Table S6). In the replication set, no significant associations between micronutrients and sepsis were detected, with the relationship between β-carotene and sepsis in the opposite direction compared to the discovery set (*p* = 0.119, IVW). Following the meta-analysis, a nominal significant correlation was found solely between Fe and the susceptibility to sepsis infection (odds ratio [OR] = 1.083; 95% confidence interval [CI]: 1.00, 1.17; *p* = 0.048) (Figure 2; Supplementary Table S5).

The risk of sepsis showed no significant correlation with the levels of calcium, β-carotene, magnesium, phosphorus, vitamin B6, vitamin C, vitamin D, and Zn in the bloodstream (Figure 2; Supplementary Table S5).

### Secondary MR analyses

**FIGURE 3:**
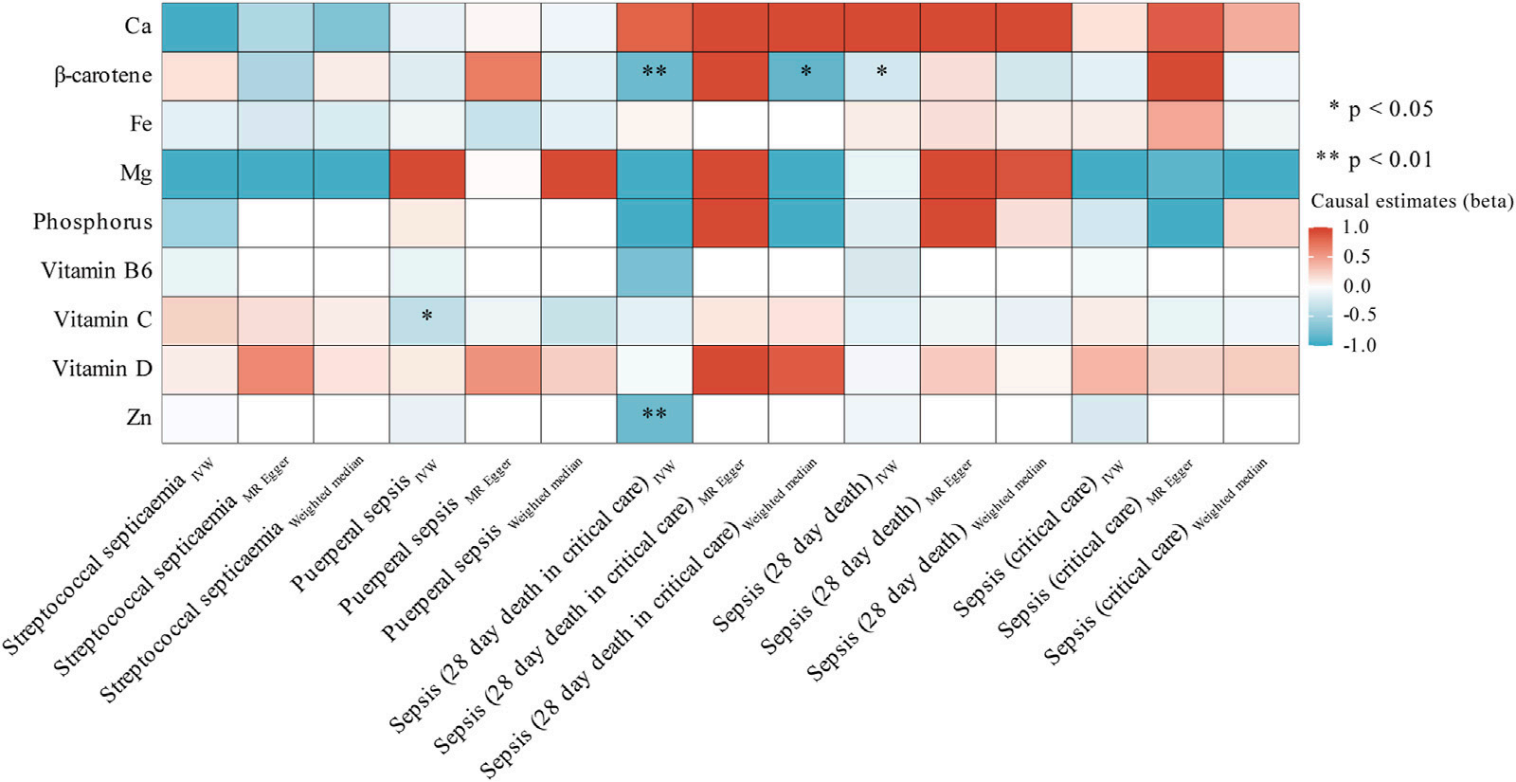
Heatmap showing the causal effects of circulating micronutrients levels on the risk of sepsis-related outcomes by using three method (IVW, MR egger, and weighted median). Abbreviations: Ca, Calcium; Fe, iron, Mg, Magnesium; Zn, zinc. IVW, inverse-variance weighted.

In subgroup analyses, we observed associations between three micronutrients and three sepsis-related outcomes. As shown in Figure 3; Supplementary Table S1, we found a nominal significant negative relationship between β-carotene and the likelihood of sepsis death within 28 days (OR = 0.781; 95% CI: 0.611, 0.997; *p* = 0.047, IVW) and severe sepsis-related death within 28 days (OR = 0.449; 95% CI: 0.253, 0.799; *p* = 6.48 × 10^−3^, IVW). The sensitivity analyses (Supplementary Tables S7, S8), which involved the use of Cochran’s Q test and I^2^ values, indicated the absence of heterogeneity. Additionally, the MR-Egger analysis, with a small intercept, showed minimal influence of horizontal pleiotropy. Likewise, the MR-PRESSO examination did not detect any unusual SNPs or horizontal pleiotropy impacts on sepsis death within 28 days (*p* = 0.958) or severe sepsis-related death within 28 days (*p* = 0.64) (Supplementary Table S9). However, the MR-Egger method shows a direction opposite to IVW, and it did not pass our stringent significance threshold. Simultaneously, we also observed a nominal significant negative association between genetically predicted vitamin C and a reduced risk of puerperal sepsis (OR = 0.702; 95% CI: 0.507, 0.971; *p* = 0.032, IVW). Although sensitivity analysis found no evidence of heterogeneity or pleiotropy, it still did not meet our stringent statistical threshold.

In contrast, there was a strong correlation between the level of Zn and a decreased likelihood of severe sepsis-related death within 28 days (OR = 0.450; 95% CI: 0.263, 0.770; *p* = 3.58 × 10^−3^, IVW). Supplementary Table S8 presents the outcomes of sensitivity analyses. Cochran’s Q test indicated no heterogeneity (*p* = 0.339); however, the restricted number of accessible SNPs (only 2) prevented the execution of MR-PRESSO and Egger regression analyses. Moreover, we employed the Steiger test to validate whether the identified causal relationships were influenced by reverse causation. The results of Steiger do not support the existence of reverse causal effects between candidate micronutrient (β-carotene, vitamin C, and Zn) and sepsis-related outcomes (Supplementary Table S9).

Given the limited availability of only two SNPs for the micronutrient Zn within the threshold of *p* < 5 × 10^−8^, which precluded heterogeneity and pleiotropy tests, we conducted a *post hoc* MR analysis. In this analysis, we included variants at a more liberal threshold (*p* < 5 × 10^−7^). As shown in Supplementary Tables S2, S10, the results aligned closely with our initial findings (OR = 0.483; 95% CI: 0.298, 0.783; *p* = 3.16 × 10^−3^, IVW), and the *p*-values for heterogeneity and pleiotropy were both above 0.05, suggesting a minimal likelihood of heterogeneity and pleiotropy.

### Confounder and multivariable MR analyses

Although sensitivity analyses did not uncover any indications of bias that would make the MR estimates unreliable, we proceeded to examine the second characteristic linked to the leading SNP for Zn by utilizing the PhenoScanner tool [blood metabolites (Wei et al., 2023), BMI (Wang et al., 2023), insomnia (Thorkildsen et al., 2023), and lifetime smoking (Zhu et al., 2023)]. However, no connections were observed between Zn-related instrumental variables and reported risk factors (Supplementary Table S11). Nonetheless, it is noteworthy that Zn’s rs1532423 was closely associated with mean corpuscular hemoglobin concentration (GWAS identifier: ebi-a-GCST90002328), reticulocyte count (GWAS identifier: ebi-a-GCST90025972), high light scatter reticulocyte count (GWAS identifier: ebi-a-GCST90025970), reticulocyte fraction of red cells (GWAS identifier: ebi-a-GCST90002406), and mean corpuscular volume (GWAS identifier: ebi-a-GCST90025963). Hence, we performed a multivariable MR analysis to examine the association between Zn and potential pleiotropic traits and the likelihood of severe sepsis-related death within 28 days.

After accounting for the impacts of mean corpuscular hemoglobin concentration, reticulocyte count, high light scatter reticulocyte count, reticulocyte fraction of red cells, and mean corpuscular volume in the multivariable MR analysis, we found comparable effects to the primary analysis, suggesting that Zn continued to exhibit a defensive influence on severe sepsis-related death within 28 days (OR = 0.675; 95% CI: 0.508, 0.895; *p* = 6.421 × 10^−3^, IVW) (Table 3).

**TABLE 3:** Estimated causal effects of zinc on Sepsis (28 day death in critical care) by the multivariable Mendelian randomization analysis.

Exposure		Multivariable MR	
	Nsnp	OR (95% CI)	*P*
Mean corpuscular hemoglobin concentration	227	1.537 (0.601, 3.932)	0.370
Reticulocyte fraction of red cells	227	0.335 (0.010, 11.218)	0.541
Mean corpuscular volume	227	0.921 (0.514, 1.652)	0.783
High light scatter reticulocyte count	227	6.106 (0.839, 44.454)	0.074
Reticulocyte count	227	0.462 (0.011, 19.751)	0.687
Zinc	227	0.675 (0.508, 0.895)	6.421E-03

Abbreviations: OR, odds ratio; CI, confidence interval, Nsnp, number of SNP.

### Reverse MR analyses

In order to further explore the causal efficacy connection between potential micronutrients and outcomes related to sepsis, we performed reverse causal analyses by employing instrumental variables for sepsis-related outcomes. Our objective was to use IVW-MR estimates and select significant independent SNPs with a *p* < 5 × 10^−8^ as instrumental variables. This analysis aimed to investigate whether there was any indication of a reverse causal association between the identified Zn and the outcome of severe sepsis-related death within 28 days. As there were no significant independent SNPs identified when considering severe sepsis-related death within 28 days as the variable at a significance level of *p* < 5 × 10^−8^ or *p* < 5 × 10^−7^, we adjusted the criteria to *p* < 5 × 10^−6^ and included linkage disequilibrium with r^2^ < 0.001 within 10,000-kb windows. Nevertheless, our examination revealed restricted backing for this inverse causal connection (beta = −0.014; 95% CI: −0.115, 0.087; *p* = 0.783, IVW), as specified in Supplementary Table S12.

## Discussion

Drawing on our current knowledge, this study represents the initial comprehensive examination of causal connections between various circulating micronutrients in the blood and the susceptibility, severity, and subtype-specific risks of sepsis. Our research findings indicate one strong correlation and four suggestive associations among four micronutrients and sepsis-related outcomes. In particular, our main finding suggests a strong causal effect connection between genetically forecasted Zn levels in the bloods and a decreased risk of severe sepsis-related death within 28 days. Additionally, four suggestive associations were identified: elevated blood Fe levels indicating a potential link to increased susceptibility to sepsis, higher blood β-carotene levels suggestively associated with decreased risk of severe sepsis-related death within 28 days and sepsis-related death within 28 days, and a suggestively correlation between vitamin C and decreased risk of postpartum sepsis. There is no apparent association between the other five circulating micronutrients and sepsis or related outcomes.

Sepsis is an illness resulting from an infection, which causes dysfunction of organs and ultimately leads to death. Sepsis is a significant worldwide contributor to death, causing approximately 6 million fatalities each year (Evans et al., 2021). Timely identification and medical intervention are vital for individuals who might be susceptible to septicemia. In recent years, the role of circulating micronutrients in blood in disease has garnered increasing attention. Zn, a micronutrient, has been demonstrated to be a vital metal ion for a well-functioning immune system. In the human body, it has a vital function in cellular differentiation, proliferation, and apoptosis mechanisms. According to Gammoh’s research (Gammoh and Rink, 2017), Zn has the ability to control the discharge of inflammatory substances, the production of coenzymes, and the operation of T helper cells, B cells, neutrophils, natural killer cells, and macrophages.

Previous studies have found an association between Zn deficiency and compromised immune function, as well as adverse disease outcomes (Overbeck et al., 2008). Low-dose Zn supplementation has been shown to effectively treat respiratory infections and childhood diarrhea (Dhingra et al., 2020). However, the connection between Zn levels in blood and the risk of human sepsis infection has not been clearly established. Research has observed significantly lower serum Zn concentrations in ICU sepsis patients compared to healthy controls (Hoeger et al., 2017). However, a randomized controlled trial found no notable distinctions between the group that received Zn supplementation and the control group among sepsis patients (Mehta et al., 2013), and it even indicated potential adverse consequences (Braunschweig et al., 1997). The uncertain outcomes could be impacted by methodological deficiencies like limited sample sizes or remaining confounding factors. From a genetic standpoint, our MR study presents proof that genetically anticipated Zn concentrations in the bloodstream offer a safeguarding influence on the severe sepsis-related death within 28 days, despite the fact that the influence of Zn on sepsis and severe sepsis is limited. An animal experiment has identified a potential mechanism behind these findings: Zn can modulate host immune defense by blocking the IKK complex and inducing inhibition of the NF-κB pathway downstream of MAPK (Liu et al., 2013). However, supplementing Zn during infection needs to consider the risk of creating a Zn microenvironment favorable to pathogen growth while interfering with the innate immune system’s ability to chelate free Zn. Our study may suggest that, although Zn is not associated with the risk of severe sepsis, it supports clinical practices of Zn supplementation in critically ill sepsis patients at high risk of mortality. Nevertheless, additional medical investigation is required to authenticate these discoveries.

We additionally discovered a slight positive correlation between genetically anticipated blood Fe levels and the susceptibility of sepsis. Consistent with previous observational studies on sepsis, septic patients had higher serum iron levels compared to healthy volunteers (Akkas et al., 2020). Fe is an essential element in various physiological processes, and deviations in Fe status (such as Fe deficiency or Fe overload) can significantly impact health. Fe status deviation exhibits noticeable gender differences, with females being more prone to Fe deficiency. Prior observational studies have suggested associations between both iron deficiency (Mohus et al., 2018) and high iron status (Brandtner et al., 2020) with an increased risk of infection. According to a recent study using magnetic resonance imaging (MRI), it was found that the addition of Fe is not likely to greatly raise the chances of infection (Butler-Laporte et al., 2023). Conversely, a separate study using the same method indicated a positive association between the predicted increase in serum Fe levels and an elevated risk of sepsis (Mohus et al., 2022). Unfortunately, the latter study only set the significance threshold at *p* < 0.05 without rigorous correction for multiple testing. It is worth noting that the observed associations do not imply a strong causal relationship, and the impact of Fe on sepsis appears to be relatively mild. Future research endeavors should explore this relationship further, conducting rigorous analyses to validate these findings.

Regarding β-carotene, long considered potent antioxidants within the organism, prior epidemiological studies have proposed a negative correlation between carotenoid intake and cancer incidence (Koklesova et al., 2020). Moreover, cancer patients exhibit a significant increase in carotenoid concentrations after anti-tumor treatment (McMillan et al., 2000). A recent MR study found a protective effect of blood β-carotene against type 2 diabetes (Chen et al., 2023).These findings collectively support the beneficial role of β-carotene in disease occurrence. In our research, we noticed a slight adverse correlation between β-carotene and the likelihood of severe sepsis as well as the mortality rate within 28 days for individuals with severe sepsis. Nevertheless, this discovery could be fortuitous as a result of conducting numerous tests and did not meet our rigorous statistical significance criteria.

Likewise, we observed a suggestive causal effect of higher circulating levels of vitamin C in reducing the risk of puerperal sepsis. Previous observational studies have noted significantly decreased average vitamin C levels in sepsis patients (Carr et al., 2017), prompting discussions on the potential therapeutic role of vitamin C as a crucial antioxidant in sepsis management. A recent review, considering findings from conducted randomized controlled trials, reported positive effects of vitamin C on reducing sepsis mortality in only 2 out of 11 projects (Ammar et al., 2021). Some studies suggest that vitamin C, compared to a placebo, may contribute to mitigating inflammation induced by severe sepsis (Fowler et al., 2014). However, a recent randomized controlled trial yielded inconsistent results, showing that vitamin C did not significantly improve sepsis-related inflammation and vascular damage (Fowler et al., 2019). The heterogeneity in vitamin C treatment regimens, initiation times, and duration of therapy has led to significant variability in results across observational studies. Our MR study did not find causal effects of vitamin C on susceptibility and severity of sepsis at genetic level. However, it revealed a mild protective effect of vitamin C specifically in one subtype of sepsis—postpartum sepsis. Nevertheless, this finding did not meet our stringent statistical thresholds, and given the limited observational studies on postpartum sepsis to date, larger-scale research is needed to further explore this relationship in the future.

Surprisingly, there were no connections discovered between genetically anticipated levels of calcium, magnesium, phosphorus, vitamin D, and vitamin B6 in the bloodstream and the likelihood of sepsis-related consequences. Contrary to a meta-analysis suggesting that vitamin D deficiency increases susceptibility and mortality in sepsis (de Haan et al., 2014). Our study does not support this viewpoint. Additionally, a prospective cohort study found insufficient evidence for vitamin D in predicting sepsis and mortality rates (Ratzinger et al., 2017). A review concluded that there is no clear evidence that selenium supplementation can prevent infection and new infection rates (Zhao et al., 2019). This may suggest that these micronutrients are not crucial risk factors for the development of sepsis and its related outcomes.

Our study has several strengths. This is the initial comprehensive study of MR that examines the association between the levels of 9 micronutrients and the risk of sepsis and its related outcomes. This helps reduce the influence of confounding factors present in observational studies. Furthermore, our examination was limited to people of European origin, reducing the occurrence of population stratification errors. Thirdly, the meta-analysis of summary data from multiple sepsis cohorts reduced random errors, enhancing credibility. However, the study also has limitations. First, some micronutrients’ instrumental variables exhibited varying degrees of low statistical power. Despite all instrumental variables having F-values greater than 10, suggesting a low probability of weak instrument bias, there is still a possibility of some bias remaining. To enhance statistical power, it is imperative to conduct future GWAS on traits related to micronutrients at a larger scale. Second, the study population limited to individuals of European descent may hinder generalization to a broader population. Third, due to sparse subtype data, we could not replicate the associations between Zn and susceptibility or severity subtypes of sepsis. However, our findings suggest a protective trend of Zn against all five sepsis-related outcomes. Larger-scale clinical studies are needed to further confirm these findings and explore underlying mechanisms.

## Conclusion

To summarize, our research indicates that Zn might have a safeguarding effect in decreasing the likelihood of death within 28 days for patients with severe sepsis, endorsing the medical recommendation of providing Zn supplements to patients who face a high risk of mortality due to severe sepsis. This provides new insights for further research into the role of micronutrients in the prevention and treatment of sepsis.

## Scope statement

Previous research has paid limited attention to the role of trace micronutrients in the pathogenesis of sepsis. Therefore, we employed Mendelian randomization analysis to comprehensively investigate the causal relationship between the levels of nine micronutrients (including calcium, β-carotene, iron, magnesium, phosphorus, vitamin C, vitamin B6, vitamin D, and zinc) and susceptibility, severity, and subtypes of sepsis. Our research indicates that zinc might have a safeguarding effect in decreasing the likelihood of death within 28 days for patients with severe sepsis, endorsing the medical recommendation of providing zinc supplements to patients who face a high risk of mortality due to severe sepsis. This provides new insights for further research into the role of micronutrients in the prevention and treatment of sepsis.

## Data Availability

The original contributions presented in the study are included in the article/Supplementary Material, further inquiries can be directed to the corresponding author.
