# Supplementary material for: Circulating micronutrient levels and their association with sepsis susceptibility and severity: a Mendelian randomization study
Source: Front Genet. 2024 Feb 16;15:1353118. doi: 10.3389/fgene.2024.1353118 (PMC10904592; doi:10.3389/fgene.2024.1353118)
Supplement: Supplementary file 1 [file Table1.docx]

***Supplementary Material***

**Circulating micronutrient Levels and their association with sepsis susceptibility and severity: a Mendelian randomization study**

Zhengxiao Wei^1^, Yingfen Liu^1^, Xue Mei^2^, Jing Zhong^1^ and Fuhong Huang^3^*

^1^ Department of Clinical Laboratory, Public Health Clinical Center of Chengdu, Chengdu, Sichuan, China.

^2^ Department of Infectious Diseases, Public Health Clinical Center of Chengdu, Chengdu, Sichuan, China.

^3^ Department of Ultrasound, Sichuan Provincial People’s Hospital, University of Electronic Science and Technology of China, Chengdu, China.

***Corresponding author**: Fuhong Huang;

Email: fuhong_huang@163.com

**Short title:** Micronutrients and risk of sepsis

**Keywords:** Micronutrients, Mendelian randomization, Sepsis, Susceptibility, Zinc

**Supplementary Figure S1.** Forest plot for the meta-analysis of circulating micronutrients levels on the risk of sepsis-related outcomes by using IVW method.

Abbreviations: IVW, inverse-variance weighted; Nsnp, number of SNP; OR, odds ratio; CI, confidence interval; Ca, Calcium; Fe, iron, Mg, Magnesium; Zn, zinc.

**
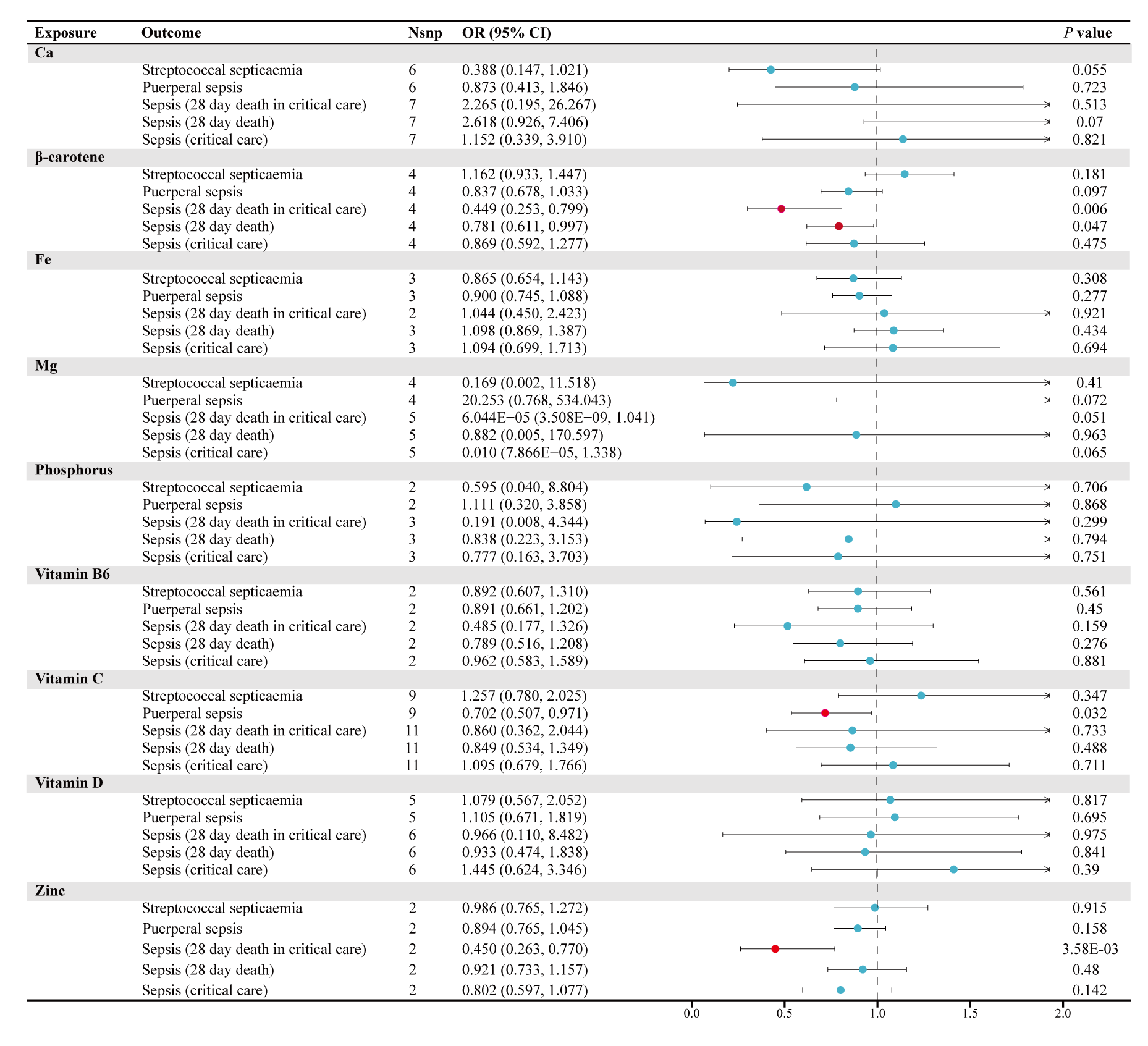
**

**Supplementary Figure S2.** Funnel plot and scatter plot of MR analysis of zinc as protective factors on the risk of Sepsis (28 day death in critical care)

**
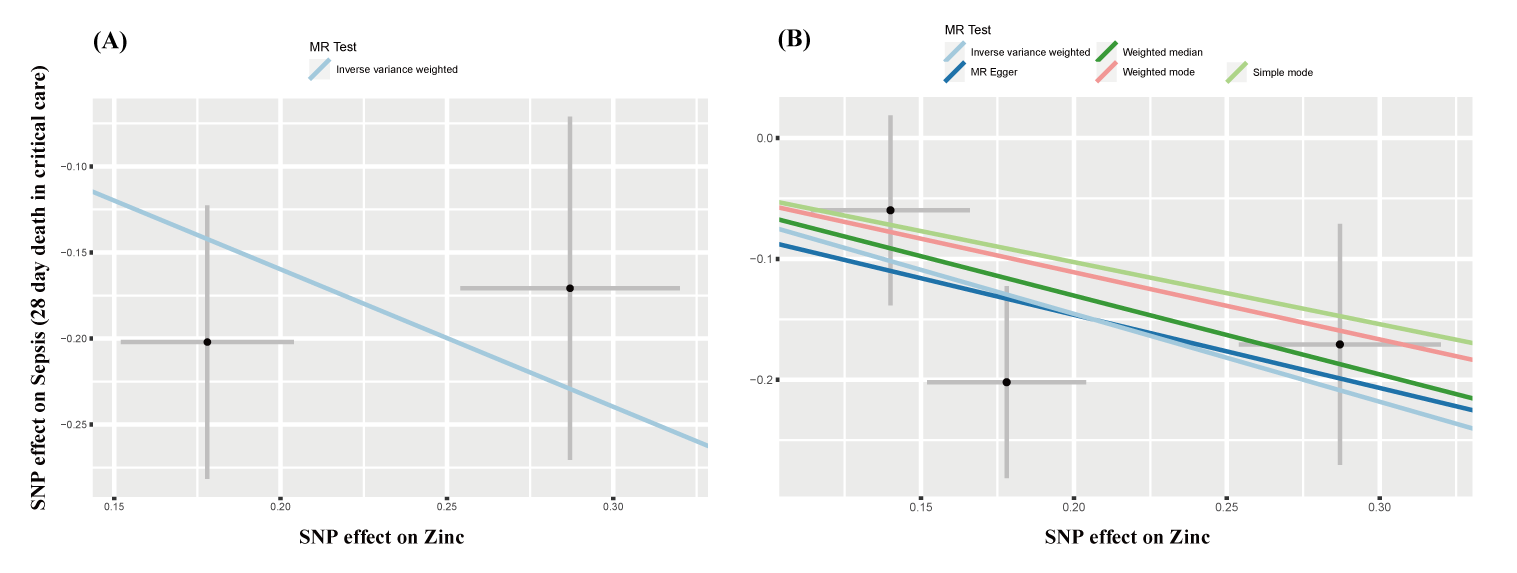
**

**Supplementary Table S1.** STROBE-MR checklist of recommended items to address in reports of Mendelian randomization studies

| **Item No.** | **Section** | **Checklist item** | **Section (paragraph number)** |
| --- | --- | --- | --- |
| 1 | **TITLE and ABSTRACT** | Indicate Mendelian randomization as the study’s design in the title and/or the abstract if that is a main purpose of the study | Title page |
|  | **INTRODUCTION** |  |  |
| 2 | **Background** | Explain the scientific background and rationale for the reported study. What is the exposure? Is a potential causal relationship between exposure and outcome plausible? Justify why MR is a helpful method to address the study question | Introduction (paragraphs 1-4) |
| 3 | **Objectives** | State specific objectives clearly, including pre-specified causal hypotheses (if any). State that MR is a method that, under specific assumptions, intends to estimate causal effects | Introduction (paragraph 4), Methods (paragraph 1) |
|  | **METHODS** |  |  |
| 4 | **Study design and data sources** | Present key elements of the study design early in the article. Consider including a table listing sources of data for all phases of the study. For each data source contributing to the analysis, describe the following: | Methods (paragraphs 1-11), Supplementary Table S2 |
|  | a) | Setting: Describe the study design and the underlying population, if possible. Describe the setting, locations, and relevant dates, including periods of recruitment, exposure, follow-up, and data collection, when available. | Methods (paragraphs 1-5), Table 1-2, Supplementary Table S2 |
|  | b) | Participants: Give the eligibility criteria, and the sources and methods of selection of participants. Report the sample size, and whether any power or sample size calculations were carried out prior to the main analysis | Methods (paragraphs 1-5, 10), Table 1-2, Supplementary Table S2 |
|  | c) | Describe measurement, quality control and selection of genetic variants | Methods (paragraph 6) |

*Continued on next page*

**Supplementary Table S1. *Continued***

|  | d) | For each exposure, outcome, and other relevant variables, describe methods of assessment and diagnostic criteria for diseases | Methods (paragraphs 3-6) |
| --- | --- | --- | --- |
|  | e) | Provide details of ethics committee approval and participant informed consent, if relevant | Methods (paragraph 2) |
| 5 | **Assumptions** | Explicitly state the three core IV assumptions for the main analysis (relevance, independence and exclusion restriction) as well assumptions for any additional or sensitivity analysis | Methods (paragraph 7) |
| 6 | **Statistical methods: main analysis** | Describe statistical methods and statistics used |  |
|  | a) | Describe how quantitative variables were handled in the analyses (i.e., scale, units, model) | Methods (paragraph 10,12) |
|  | b) | Describe how genetic variants were handled in the analyses and, if applicable, how their weights were selected | Methods (paragraphs 6,10-12) |
|  | c) | Describe the MR estimator (e.g. two-stage least squares, Wald ratio) and related statistics. Detail the included covariates and, in case of two-sample MR, whether the same covariate set was used for adjustment in the two samples | Methods (paragraph 7-9) |
|  | d) | Explain how missing data were addressed | Methods (paragraphs 6-8) |
|  | e) | If applicable, indicate how multiple testing was addressed | Methods (paragraphs 12) |
| 7 | **Assessment of assumptions** | Describe any methods or prior knowledge used to assess the assumptions or justify their validity | Methods (paragraphs 6-9,11) |
| 8 | **Sensitivity analyses and additional analyses** | Describe any sensitivity analyses or additional analyses performed (e.g. comparison of effect estimates from different approaches, independent replication, bias analytic techniques, validation of instruments, simulations) | Methods (paragraphs 7-9,11) |

*Continued on next page*

**Supplementary Table S1. *Continued***

| 9 | **Software and pre-registration** |  |  |
| --- | --- | --- | --- |
|  | a) | Name statistical software and package(s), including version and settings used | Methods (paragraph 12) |
|  | b) | State whether the study protocol and details were pre-registered (as well as when and where) | N/A |
|  | **RESULTS** |  |  |
| 10 | **Descriptive data** |  |  |
|  | a) | Report the numbers of individuals at each stage of included studies and reasons for exclusion. Consider use of a flow-diagram | Figure 1, Table 1-2, Supplementary Table S2 |
|  | b) | Report summary statistics for phenotypic exposure(s), outcome(s) and other relevant variables (e.g. means, SDs, proportions) | N/A |
|  | c) | If the data sources include meta-analyses of previous studies, provide the assessments of heterogeneity across these studies | N/A |
|  | d) | For *two-sample* Mendelian randomisation:  i.  Provide justification of the similarity of the genetic variant-exposure associations between the exposure and outcome samples  ii.  Provide information on the number of individuals who were in both samples for the exposure and for the outcome | N/A |

*Continued on next page*

**Supplementary Table S1. *Continued***

| 11 | **Main results** |  |  |
| --- | --- | --- | --- |
|  | a) | Report the associations between genetic variant and exposure, and between genetic variant and outcome, preferably on an interpretable scale | Results (paragraph 2-5), Figure 2-3 |
|  | b) | Report MR estimates of the relationship between exposure and outcome, and the measures of uncertainty from the MR analysis, on an interpretable scale, such as odds ratio or relative risk per SD difference | Results (paragraph 2-5), Figure 2-3 |
|  | c) | If relevant, consider translating estimates of relative risk into absolute risk for a meaningful time period | N/A |
|  | d) | Consider plots to visualize results (e.g. forest plot, scatterplot of associations between genetic variants and outcome versus between genetic variants and exposure) | Figures 2-3, Supplementary Figures S1 |
| 12 | **Assessment of assumptions** |  |  |
|  | a) | Report the assessment of the validity of the assumptions | Results (paragraphs 2-5) |
|  | b) | Report any additional statistics (e.g., assessments of heterogeneity across genetic variants, such as I^2^, Q statistic or E-value) | Results (paragraphs 2-5), Supplementary Table S6-S8 |
| 13 | **Sensitivity analyses and additional analyses** |  |  |
|  | a) | Report any sensitivity analyses to assess the robustness of the main results to violations of the assumptions | Results (paragraphs 2-7) |
|  | b) | Report results from other sensitivity analyses or additional analyses | Results (paragraphs 2-9) |

*Continued on next page*

**Supplementary Table S1. *Continued***

|  | c) | Report any assessment of direction of causal relationship (e.g., bidirectional MR) | Results (paragraphs 5-8) |
| --- | --- | --- | --- |
|  | d) | When relevant, report and compare with estimates from non-MR analyses | N/A |
|  | e) | Consider additional plots to visualize results (e.g., leave-one-out analyses) | Figures 2-3, Supplementary Figures S1 |
|  | **DISCUSSION** |  |  |
| 14 | **Key results** | Summarize key results with reference to study objectives | Discussion (paragraph 1) |
| 15 | **Limitations** | Discuss limitations of the study, taking into account the validity of the IV assumptions, other sources of potential bias, and imprecision. Discuss both direction and magnitude of any potential bias and any efforts to address them | Discussion (paragraph 8) |
| 16 | **Interpretation** |  |  |
|  | a) | Meaning: Give a cautious overall interpretation of results in the context of their limitations and in comparison with other studies | Discussion (paragraph 2-7) |
|  | b) | Mechanism: Discuss underlying biological mechanisms that could drive a potential causal relationship between the investigated exposure and the outcome, and whether the gene-environment equivalence assumption is reasonable. Use causal language carefully, clarifying that IV estimates may provide causal effects only under certain assumptions | Discussion (paragraphs 3) |
|  | c) | Clinical relevance: Discuss whether the results have clinical or public policy relevance, and to what extent they inform effect sizes of possible interventions | Discussion (paragraph 3-8) |
| 17 | **Generalizability** | Discuss the generalizability of the study results (a) to other populations, (b) across other exposure periods/timings, and (c) across other levels of exposure | Discussion (paragraph 3,8) |

*Continued on next page*

**Supplementary Table S1. *Continued***

|  | **OTHER INFORMATION** |  |  |
| --- | --- | --- | --- |
| 18 | **Funding** | Describe sources of funding and the role of funders in the present study and, if applicable, sources of funding for the databases and original study or studies on which the present study is based | Funding section |
| 19 | **Data and data sharing** | Provide the data used to perform all analyses or report where and how the data can be accessed, and reference these sources in the article. Provide the statistical code needed to reproduce the results in the article, or report whether the code is publicly accessible and if so, where | Data availability section |
| 20 | **Conflicts of Interest** | All authors should declare all potential conflicts of interest | Competing interests section |

| **Supplementary Table S2.** Information of exposure GWAS cohorts | |  |
| --- | --- | --- |
| **Exposure** | **The information of exposure GWAS cohorts** | **Pubmed ID** |
| Ca | The genetic instruments used for calcium were extracted from a meta-analysis conducted using 39,400 individuals from 17 population-based cohorts in the discovery stage and 21,679 individuals in the replication stage, totaling approximately 61,079 individuals. The extracted SNP was from either the discovery (n = 39,400) cohort or the meta-analysis. The mean concentration of serum calcium level was 9.47 mg/dL (SD 0.52). The covariates included in the GWAS were age, sex, and study-specific covariates, if needed, such as principal components and study center. | 24068962 |
| Cu | The genetic instruments used for copper were extracted from a GWAS conducted using 2,603 twins and families from the Queensland Institute of Medical Research (QIMR). The mean concentration of erythrocyte Cu was 43.7 nmol/g (SD 12.15). Estimates for genetic associations with log-transformed standardized residuals of copper were adjusted for sex and age via an additive model accounting for within-family relatedness, where the effect was measured in SD. | 23720494 |
| Fe | The genetic instruments used for iron were extracted from the Genetics of Iron Status Consortium (GISC), samples consisted of summary data on genome-wide allelic associations from 23,986 subjects of European ancestry gathered from 11 cohorts in 9 participating centres. The meta-analysis utilized a standard error-based approach, adjusted for age, principal component scores, and other study-specific covariates, and the effect was measured in SD. | 25352340 |

*Continued on next page*

**Supplementary Table S2. *Continued***

| Mg | The genetic instruments used for magnesium were extracted from a meta-analysis using approximately 15,366 individuals from the Cohorts for Heart and Aging Research in Genomic Epidemiology (CHARGE) consortium, which consisted of 8,122 individuals from the Atherosclerosis Risk in Communities (ARIC) Study, 2,866 individuals from the Framingham Heart Study (FHS) and 4,378 individuals from the Rotterdam Study (RS). The mean concentration of serum magnesium was 0.84 mmol/L (SD 0.09). The individual GWASs were adjusted for age, sex, and study center (if applicable), and meta-analyses were performed using inverse-variance weighted fixed-effects models. | 20700443 |
| --- | --- | --- |
| Phosphorus | The genetic instruments used for phosphorus were extracted from a meta-analysis using approximately 21,733 individuals from four discovery cohorts [1,761 individuals from the Cardiovascular Health Study (CHS), 2,865 individuals from the Framingham Heart Study (FHS), 8,048 individuals from the Atherosclerosis Risk in Communities Study (ARIC), and 3,516 individuals from the Rotterdam Study (RS)] and four replication cohorts [1559 individuals from The Cooperative Health Research in the Region of Augsburg (KORA) F3 , 1,779 individuals from KORA-F4, 1,507 individuals from Health ABC, and 659 individuals from Vis ]. The mean concentration of serum phosphorus was 3.5 mg/dL (SD 0.31). The data were combined using fixed-effect meta-analyses based on P values and direction of effect adjusted for age, sex, and study site. | 20558539 |
| Se | The genetic instruments used for selenium were extracted from a meta-analysis using 9,639 european descendants sourced from six independent population-based studies: Coronary Artery Risk Development in Young Adults (CARDIA), Johnston County Osteoarthritis Project (JoCo), Nurses' Health Study (NHS), the Health Professionals Follow-up Study (HPFS), Queensland Institute of Medical Research (QIMR) study and The Avon Longitudinal Study of Parents and Children (ALSPAC) cohort. Analyses were stratified by gender and adjusted for age, smoking status [never/former, current (<15 cig/day), current >or=15 cig/day)], geographical area of residence (NHS, HPFS, JoCo) or study site (CARDIA) and top eigenvectors (number varies by data set). | 25343990 |

*Continued on next page*

**Supplementary Table S2. *Continued***

| Zn | The genetic instruments used for zinc were extracted from a GWAS conducted using 2,603 individuals from the QIMR cohort. Estimates for genetic associations with log-transformed standardized residuals of zinc were adjusted for sex and age via an additive model accounting for within-family relatedness, where the effect was measured in SD. | 23720494 |
| --- | --- | --- |
| Folate | The genetic instruments used for folate were extracted from GWAS conducted using 28,913 Icelanders. The mean concentration of serum folate was 22.6 pmol/L (SD 11.33) for Icelanders. The GWAS was adjusted for sex, year of birth, and age at measurement. | 23754956 |
| β-carotene | The genetic instruments used for beta-carotene were extracted from a meta-analysis conducted using 1,190 individuals from the Invecchiare in Chianti (InCHIANTI) study, 576 individuals from Women's Health and Aging Study (WHAS), and 2,126 individuals from the α-Tocopherol, β-Carotene Cancer Prevention (ATBC) Study, totaling approximately 3,892 individuals. The mean concentration serum/plasma level of beta-carotene was 0.41 μmol/L (SD 0.27). The meta-analysis was performed across different studies by weighting each with an inverse variance method. The individual GWASs were adjusted for age and sex. | 19185284 |
| Vitamin B6 | The genetic instruments used for vitamin B6 were extracted from a meta-analysis using 1,658 individuals from the Nurses’ Health Study (NHS) Cancer Genetic Markers of Susceptibility (CGEMS), 1,647 from Framingham-SNP-Health Association Resource (SHARe) Women, and 1,458 from SHARe men, totaling approximately 4,763 individuals. The mean concentration of plasma vitamin B6 was 81.94 pmol/mL (SD 86.03). A fixed-effect model was used to conduct the meta-analysis. The CGEMS GWAS was adjusted for age and principal components, while the SHARe data was adjusted for age. | 19744961 |

*Continued on next page*

**Supplementary Table S2. *Continued***

| Vitamin B12 | The genetic instruments used for vitamin B12 were extracted from a meta-analysis using approximately 25,960 individuals from Icelandic, 5,481 individuals from Danish – inter99, and 2,812 individuals from Danish -Health 2006 study, totaling 45,575. The mean concentration of serum vitamin B12 was 385.07 pmol/L (SD 221.09). The data were combined using fixed-effect meta-analyses based on P values and direction of effect adjusted for the number of individuals in each sample. | 23754956 |
| --- | --- | --- |
| Vitamin C | The genetic instruments used for vitamin C were extracted from a meta-analysis of 52,018 individuals using four cohort studies, Fenland, European Prospective Investigation into Cancer (EPIC) - Norfolk, EPIC-InterAct, and EPIC Cardiovascular disease (CVD). The mean concentration of plasma vitamin C was 50.1 umol/L (SD 23.1). The meta-analysis of GWAS results was conducted by combining beta coefficients and SE using inverse variance-weighted fixed-effect meta-analysis across the participating studies. The individual GWASs were adjusted for age, sex, study center (where appropriate), and the first ten principal components. | 33203707 |
| Vitamin D | The genetic instruments used for vitamin D were extracted from GWAS conducted using 122,123 individuals from 31 cohorts in Europe, Canada and USA. The GWAS was performed on standardized log-transformed vitamin D expressed in nmol/L, adjusted the models for month of sample collection (12 categories), age, sex, and body mass index, and principal components capturing genetic ancestry. | 29343764 |

Abbreviations: Ca, Calcium; Cu, Copper; Fe, iron, Mg, Magnesium; Se, Selenium; Zn, zinc.

| **Supplementary Table S3.** Power calculations. | | | | | | |
| --- | --- | --- | --- | --- | --- | --- |
| **Exposure** | **Sepsis** | **Sepsis (critical care)** | **Sepsis (28 day death)** | **Sepsis (28 day death in critical care)** | **Streptococcal septicaemia** | **Puerperal sepsis** |
| Ca | 10 | 8 | 100 | 58 | 78 | 11 |
| Cu | 19 | 7 | 36 | 29 | 32 | 6 |
| Fe | 58 | 9 | 12 | 5 | 21 | 19 |
| Mg | 34 | 99 | 10 | 62 | 100 | 100 |
| Phosphorus | 81 | 15 | 12 | 38 | 58 | 12 |
| Se | 8 | 21 | 8 | 9 | 7 | 7 |
| Folate | 19 | 20 | 14 | 14 | 6 | 11 |
| β-carotene | 58 | 29 | 79 | 84 | 62 | 84 |
| Vitamin B6 | 80 | 9 | 36 | 39 | 0.15 | 22 |
| Vitamin B12 | 10 | 6 | 9 | 6 | 21 | 7 |
| Vitamin C | 5 | 8 | 14 | 6 | 38 | 71 |
| Vitamin D | 7 | 77 | 8 | 5 | 10 | 19 |
| Zn | 7 | 35 | 11 | 59 | 5 | 30 |
| Results are presented in % | | | | | | |
| Abbreviations: Ca, Calcium; Cu, Copper; Fe, iron, Mg, Magnesium; Se, Selenium; Zn, zinc. | | | | | | |

| **Supplementary Table S4.** Genetic variants used as exposure for Mendelian randomization analyses | | | | | | | | | | | | | |
| --- | --- | --- | --- | --- | --- | --- | --- | --- | --- | --- | --- | --- | --- |
| **Exposure** | **SNPs** | **CHR** | **BP** | **Genes** | **EA** | **OA** | **N** | **EAF** | **Beta** | **StdErr** | **P value** | **R^2^** | **F statistics** |
| Ca | rs1801725 | 3 | 122284910 | *CASR* | T | G | 61054 | 0.15 | 0.071 | 0.004 | 8.90E-86 | 0.005134 | 315 |
|  | rs1550532 | 2 | 233356202 | *DGKD* | C | G | 60998 | 0.31 | 0.018 | 0.003 | 8.20E-11 | 0.00059 | 36 |
|  | rs780094 | 2 | 27518370 | *GCKR* | T | C | 60958 | 0.42 | 0.017 | 0.003 | 1.30E-10 | 0.000526 | 32 |
|  | rs10491003 | 10 | 9286688 | *GATA3* | T | C | 60040 | 0.09 | 0.027 | 0.005 | 4.80E-09 | 0.000485 | 29 |
|  | rs7481584 | 11 | 3007859 | *CARS* | A | G | 61011 | 0.3 | -0.018 | 0.003 | 1.20E-10 | 0.00059 | 36 |
|  | rs7336933 | 13 | 41984940 | *DGKH; KIAA0564* | A | G | 60928 | 0.15 | -0.022 | 0.004 | 9.10E-10 | 0.000496 | 30 |
|  | rs1570669 | 20 | 54157888 | *CYP24A1* | A | G | 60966 | 0.66 | -0.018 | 0.003 | 9.10E-12 | 0.00059 | 36 |
| β-carotene | rs6420424 | 16 | 81208497 | *PKD1L2* | G | A | 1793 | 0.05 | 0.155 | 0.022 | 6.50E-13 | 0.026939 | 50 |
|  | rs8044334 | 16 | 81215330 | *PKD1L2* | T | G | 3915 | 0.33 | 0.109 | 0.015 | 9.30E-13 | 0.013308 | 53 |
|  | rs11645428 | 16 | 81225291 | *PKD1L2* | G | A | 3918 | 0.35 | -0.129 | 0.015 | 1.50E-17 | 0.018527 | 74 |
|  | rs6564851 | 16 | 81230992 | *BCO1* | T | G | 3881 | 0.49 | 0.149 | 0.015 | 1.60E-24 | 0.024794 | 99 |
| Fe | rs1525892 | 3 | 133765868 | *TF* | A | G | 23986 | 0.652 | 0.0736 | 0.0104 | 1.65E-12 | 0.002084 | 50 |
|  | rs1800562 | 6 | 26092913 | *HFE* | A | G | 23986 | 0.043 | 0.3724 | 0.02 | 3.96E-77 | 0.014248 | 347 |
|  | rs855791 | 22 | 37066896 | *TMPRSS6* | G | A | 23986 | 0.612 | 0.1868 | 0.0101 | 4.31E-77 | 0.014061 | 342 |
| Mg | rs4072037 | 1 | 155192276 | *MUC1* | C | T | 23829 | 0.46 | -0.01 | 0.001 | 2.01E-36 | 0.004179 | 100 |
|  | rs13146355 | 4 | 76490987 | *SHROOM3* | G | A | 23829 | 0.56 | -0.005 | 0.001 | 6.27E-13 | 0.001048 | 25 |
|  | rs11144134 | 9 | 74884880 | *TRPM6* | T | C | 23829 | 0.92 | -0.011 | 0.001 | 8.21E-15 | 0.005052 | 121 |
|  | rs3925584 | 11 | 30738788 | *DCDC5* | C | T | 23829 | 0.45 | -0.006 | 0.001 | 5.20E-16 | 0.001508 | 36 |
|  | rs7965584 | 12 | 89912002 | *ATP2B1* | G | A | 23829 | 0.29 | -0.007 | 0.001 | 1.05E-16 | 0.002052 | 49 |
| Phosphorus | rs1697421 | 1 | 21496799 | NBPF3, ALPL | A | T | 21733 | 0.49 | 0.05 | 0.005 | 7.62E-24 | 0.00458 | 100 |
|  | rs17265703 | 3 | 122329797 | *CSTA* | G | A | 21733 | 0.15 | -0.036 | 0.006 | 9.87E-10 | 0.001654 | 36 |
|  | rs9469578 | 6 | 33738702 | *IP6K3* | T | A | 21733 | 0.08 | -0.059 | 0.009 | 2.77E-11 | 0.001974 | 43 |
|  | rs947583 | 6 | 135812521 | *LINC00271* | C | T | 21733 | 0.29 | 0.035 | 0.005 | 1.28E-12 | 0.00225 | 49 |
|  | rs2970818 | 12 | 4497002 | *C12orf4* | A | T | 21733 | 0.09 | 0.047 | 0.008 | 2.11E-09 | 0.001586 | 35 |
| Vitamin B6 | rs1256335 | 1 | 21563893 | *ALPL* | G | A | 4763 | 0.79 | -0.14 | 0.02 | 1.40E-15 | 0.010183 | 49 |
|  | rs4654748 | 1 | 21459575 | *NBPF3* | C | T | 4763 | 0.52 | -0.1 | 0.01 | 4.30E-11 | 0.020563 | 100 |

*Continued on next page*

**Supplementary Table S4. *Continued***

| Vitamin C | rs6693447 | 1 | 2398751 | *RER1* | T | G | 52018 | 0.551 | 0.039 | 0.006 | 6.25E-10 | 0.000812 | 39 |
| --- | --- | --- | --- | --- | --- | --- | --- | --- | --- | --- | --- | --- | --- |
|  | rs13028225 | 2 | 219166533 | *SLC23A3* | T | C | 52018 | 0.857 | 0.102 | 0.009 | 2.38E-30 | 0.002463 | 133 |
|  | rs33972313 | 5 | 139379813 | *SLC23A1* | C | T | 52018 | 0.968 | 0.36 | 0.018 | 4.61E-90 | 0.007631 | 421 |
|  | rs10051765 | 5 | 177372991 | *RGS14* | C | T | 52018 | 0.342 | 0.039 | 0.007 | 3.64E-09 | 0.000596 | 36 |
|  | rs7740812 | 6 | 5286098`·9 | *#N/A* | G | A | 52018 | 0.594 | 0.038 | 0.006 | 1.88E-09 | 0.000771 | 36 |
|  | rs174547 | 11 | 61803311 | *FADS1* | C | T | 52018 | 0.328 | 0.036 | 0.007 | 3.84E-08 | 0.000508 | 30 |
|  | rs117885456 | 12 | 95855333 | *SNRPF* | A | G | 52018 | 0.087 | 0.078 | 0.012 | 1.70E-11 | 0.000812 | 50 |
|  | rs2559850 | 12 | 101699681 | *CHPT1* | A | G | 52018 | 0.598 | 0.058 | 0.006 | 6.30E-20 | 0.001793 | 84 |
|  | rs10136000 | 14 | 104787244 | *AKT1* | A | G | 52018 | 0.283 | 0.04 | 0.007 | 1.33E-10 | 0.000627 | 34 |
|  | rs56738967 | 16 | 79706644 | *LINC01229* | C | G | 52018 | 0.321 | 0.041 | 0.007 | 7.62E-10 | 0.000659 | 38 |
|  | rs9895661 | 17 | 61379228 | *BCAS3* | T | C | 52018 | 0.817 | 0.063 | 0.008 | 1.05E-14 | 0.001191 | 62 |
| Vitamin D | rs3755967 | 4 | 71743681 | *GC* | C | T | 79366 | 0.28 | -0.089 | 0.0023 | 1.00E-200 | 0.018517 | 1497 |
|  | rs10741657 | 11 | 14893332 | *CYP2R1* | A | G | 79366 | 0.4 | 0.031 | 0.0022 | 2.05E-46 | 0.002496 | 199 |
|  | rs12785878 | 11 | 71456403 | *NADSYN1/ DHCR7* | T | G | 79366 | 0.75 | 0.036 | 0.0022 | 3.80E-62 | 0.003363 | 268 |
|  | rs17216707 | 20 | 54115823 | *CYP24A1* | T | C | 79366 | 0.79 | 0.026 | 0.0027 | 8.14E-23 | 0.001167 | 93 |
|  | rs10745742 | 12 | 95964751 | *AMDHD1* | T | C | 122123 | 0.39 | 0.019 | 0.002 | 2.10E-20 | 0.000738 | 90 |
|  | rs8018720 | 14 | 39086981 | *SEC23A* | G | C | 122123 | 0.82 | -0.019 | 0.0027 | 1.11E-11 | 0.000405 | 50 |
| Zn | rs1532423 | 8 | 85356084 | *CA1* | G | A | 2603 | 0.629 | -0.178 | 0.026 | 6.40E-12 | 0.017688 | 47 |
|  | rs2120019 | 15 | 75041843 | *PPCDC* | C | T | 2603 | 0.208 | -0.287 | 0.033 | 1.55E-18 | 0.028237 | 76 |
| Only independent SNPs (r^2^ < 0.001 within 10,000 kb windows), strongly associated (*P* ≤ 5E-08) were used as genetic instruments for the exposure. Position based on GRCh38. Abbreviations: BP, base position; CHR, chromosome; EA, effect allele; EAF, effect allele frequency; N, sample size; OA, other allele; R^2^, Proportion of the explained variance; StdErr, standard error; Ca, Calcium; Mg, Magnesium; Fe, iron; Zn, zinc. | | | | | | | | | | | | | |

| **Supplementary Table S5.** Main mendelian randomization analyses of micronutrients as risk factors on the risk of sepsis. | | | | | | | | | | | |
| --- | --- | --- | --- | --- | --- | --- | --- | --- | --- | --- | --- |
| **Exposure** | **Method** | **UK Biobank** | | |  | **FinnGen R9** | | |  | **Meta-analysis** | |
|  |  | **Nsnp** | **OR (95% CI)** | ***P* value** |  | **Nsnp** | **OR (95% CI)** | ***P* value** |  | **OR (95% CI)** | ***P* value** |
| Ca | IVW | 7 | 0.879 (0.509, 1.521) | 0.645 |  | 6 | 0.995 (0.647, 1.529) | 0.980 |  | 0.949 (0.677, 1.331) | 0.761 |
|  | MR Egger | 7 | 1.221 (0.429, 1.472) | 0.724 |  | 6 | 1.089 (0.499, 1.379) | 0.840 |  | 1.135 (0.607, 2.121) | 0.692 |
|  | Weighted median | 7 | 0.894 (0.536, 1.489) | 0.666 |  | 6 | 1.054 (0.648, 1.717) | 0.831 |  | 0.975 (0.685, 1.386) | 0.886 |
| β-carotene | IVW | 4 | 0.830 (0.748, 6.921) | 4.526E-04 |  | 4 | 1.081 (0.980, 6.192) | 0.119 |  | 0.948 (0.732, 1.227) | 0.685 |
|  | MR Egger | 4 | 0.939 (0.426, 6.071) | 0.890 |  | 4 | 1.015 (0.485, 6.125) | 0.973 |  | 0.979 (0.570, 1.679) | 0.938 |
|  | Weighted median | 4 | 0.846 (0.750, 6.955) | 0.007 |  | 4 | 1.080 (0.966, 6.208) | 0.177 |  | 0.957 (0.753, 1.216) | 0.718 |
| Fe | IVW | 3 | 1.184 (1.056, 2.328) | 0.004 |  | 3 | 0.999 (0.897, 2.114) | 0.990 |  | 1.083 (1.009, 1.171) | 0.048 |
|  | MR Egger | 3 | 1.289 (0.999, 2.663) | 0.301 |  | 3 | 0.941 (0.740, 2.196) | 0.705 |  | 1.098 (0.806, 1.495) | 0.553 |
|  | Weighted median | 3 | 1.202 (1.078, 2.339) | 8.697E-04 |  | 3 | 1.027 (0.912, 2.156) | 0.661 |  | 1.118 (1.033, 1.211) | 0.00592 |
| Mg | IVW | 5 | 1.418 (0.172, 3.673) | 0.745 |  | 4 | 0.505 (0.044, 3.788) | 0.583 |  | 0.912 (0.185, 4.493) | 0.910 |
|  | MR Egger | 5 | 0.100 (4.656E-05, 3.219) | 0.588 |  | 4 | 0.076 (6.187E-06, 3.125) | 0.645 |  | 0.090 (2.34E-04, 34.274) | 0.427 |
|  | Weighted median | 5 | 1.549 (0.145, 3.545) | 0.717 |  | 4 | 0.392 (0.044, 3.459) | 0.399 |  | 0.735 (0.148, 3.653) | 0.707 |
| Phosphorus | IVW | 3 | 0.947 (0.520, 4.724) | 0.858 |  | 2 | 0.671 (0.328, 4.373) | 0.275 |  | 0.821 (0.519, 1.301) | 0.402 |
|  | MR Egger | 3 | 0.078 (0.001, 4.168) | 0.468 |  |  |  |  |  |  |  |
|  | Weighted median | 3 | 0.777 (0.381, 4.583) | 0.487 |  |  |  |  |  |  |  |
| Vitamin B6 | IVW | 2 | 0.901 (0.751, 7.080) | 0.258 |  | 2 | 0.880 (0.741, 7.044) | 0.143 |  | 0.889 (0.785, 1.008) | 0.066 |
| Vitamin C | IVW | 11 | 1.015 (0.879, 8.172) | 0.838 |  | 9 | 0.957 (0.761, 8.204) | 0.710 |  | 0.999 (0.884, 1.128) | 0.981 |
|  | MR Egger | 11 | 1.067 (0.851, 8.339) | 0.589 |  | 9 | 1.005 (0.670, 8.507) | 0.983 |  | 1.052 (0.863, 1.282) | 0.617 |
|  | Weighted median | 11 | 1.061 (0.885, 8.271) | 0.524 |  | 9 | 0.996 (0.801, 8.237) | 0.969 |  | 1.033 (0.899, 1.188) | 0.642 |
| Vitamin D | IVW | 6 | 0.963 (0.722, 12.285) | 0.796 |  | 5 | 1.004 (0.753, 12.338) | 0.978 |  | 0.983 (0.802, 1.205) | 0.871 |
|  | MR Egger | 6 | 0.947 (0.560, 12.603) | 0.850 |  | 5 | 0.746 (0.427, 12.300) | 0.377 |  | 0.846 (0.577, 1.240) | 0.391 |
|  | Weighted median | 6 | 0.939 (0.672, 12.311) | 0.710 |  | 5 | 1.007 (0.729, 12.390) | 0.967 |  | 0.973 (0.772, 1.228) | 0.819 |
| Zn | IVW | 2 | 1.109 (0.980, 5.255) | 0.100 |  | 2 | 0.965 (0.883, 5.056) | 0.440 |  | 1.028 (0.898, 1.177) | 0.691 |

Abbreviations: IVW, inverse-variance weighted; Nsnp, number of SNP; Ca, Calcium; Mg, Magnesium; Fe, iron; Zn, zinc.

**Supplementary Table S6.** Heterogeneity and pleiotropy analysis for the causal association between circulating micronutrient and sepsis

| **Outcome** | **Source of outcome** | **Exposure** | **Nsnp** | **Heterogeneity test** | | | | |  | **Pleiotropy test** | | |
| --- | --- | --- | --- | --- | --- | --- | --- | --- | --- | --- | --- | --- |
|  |  |  |  | **IVW** | |  | **MR-Egger** | |  | **MR-Egger** | | |
|  |  |  |  | **Q** | **Q_pval** |  | **Q** | **Q_pval** |  | **Intercept** | **SE** | ***P* value** |
| sepsis | FinnGen R9 | Ca | 6 | 1.879 | 0.866 |  | 1.804 | 0.772 |  | -0.003 | 0.012 | 0.798 |
|  |  | β-carotene | 4 | 0.110 | 0.991 |  | 0.082 | 0.960 |  | 0.009 | 0.052 | 0.881 |
|  |  | Fe | 3 | 1.524 | 0.467 |  | 1.155 | 0.282 |  | 0.012 | 0.021 | 0.673 |
|  |  | Mg | 4 | 5.034 | 0.169 |  | 4.635 | 0.099 |  | 0.015 | 0.037 | 0.718 |
|  |  | Phosphorus | 2 | 0.361 | 0.548 |  |  |  |  |  |  |  |
|  |  | Vitamin B6 | 2 | 0.003 | 0.960 |  |  |  |  |  |  |  |
|  |  | Vitamin C | 9 | 16.104 | 0.041 |  | 15.912 | 0.026 |  | -0.004 | 0.013 | 0.780 |
|  |  | Vitamin D | 5 | 3.386 | 0.495 |  | 1.885 | 0.597 |  | 0.015 | 0.012 | 0.308 |
|  |  | Zn | 2 | 0.003 | 0.956 |  |  |  |  |  |  |  |
|  | UK Biobank | Ca | 7 | 9.216 | 0.162 |  | 8.320 | 0.139 |  | -0.011 | 0.015 | 0.496 |
|  |  | β-carotene | 4 | 0.372 | 0.946 |  | 0.277 | 0.871 |  | -0.017 | 0.055 | 0.788 |
|  |  | Fe | 3 | 2.664 | 0.264 |  | 1.689 | 0.194 |  | -0.019 | 0.025 | 0.586 |
|  |  | Mg | 5 | 7.155 | 0.209 |  | 6.359 | 0.174 |  | 0.020 | 0.029 | 0.518 |
|  |  | Phosphorus | 3 | 2.273 | 0.321 |  | 1.037 | 0.309 |  | 0.096 | 0.088 | 0.472 |
|  |  | Vitamin B6 | 2 | 0.261 | 0.609 |  |  |  |  |  |  |  |
|  |  | Vitamin C | 11 | 7.079 | 0.718 |  | 6.768 | 0.661 |  | -0.004 | 0.008 | 0.591 |
|  |  | Vitamin D | 6 | 4.054 | 0.542 |  | 4.049 | 0.399 |  | 0.001 | 0.012 | 0.946 |
|  |  | Zn | 2 | 1.627 | 0.202 |  |  |  |  |  |  |  |

Abbreviations: IVW, inverse-variance weighted; Nsnp, number of SNP; Ca, Calcium; Mg, Magnesium; Fe, iron; Zn, zinc.

**Supplementary Table S7.** Mendelian randomization analyses of micronutrients as risk factors on the risk of sepsis-related outcomes

| **Outcome** | **Exposure** | **Inverse variance weighted** | | |  | **MR Egger** | | |  | **Weighted median** | | |
| --- | --- | --- | --- | --- | --- | --- | --- | --- | --- | --- | --- | --- |
|  |  | **Nsnp** | **OR (95% CI)** | **pval** |  | **Nsnp** | **OR (95% CI)** | **pval** |  | **Nsnp** | **OR (95% CI)** | **pval** |
| Sepsis (critical care) | Ca | 7 | 1.152 (0.339, 3.910) | 0.821 |  | 7 | 2.333 (0.248, 21.953) | 0.492 |  | 7 | 1.507 (0.357, 6.360) | 0.577 |
|  | β-carotene | 4 | 0.869 (0.592, 1.277) | 0.475 |  | 4 | 7.265 (0.816, 64.644) | 0.217 |  | 4 | 0.909 (0.634, 1.302) | 0.602 |
|  | Fe | 3 | 1.094 (0.699, 1.713) | 0.694 |  | 3 | 1.560 (0.599, 4.061) | 0.529 |  | 3 | 0.910 (0.633, 1.308) | 0.610 |
|  | Mg | 5 | 0.010 (7.866E-05, 1.338) | 0.065 |  | 5 | 0.423 (1.002E-08, 1.785E+07) | 0.928 |  | 5 | 0.118 (2.969E-04, 47.102) | 0.485 |
|  | Phosphorus | 3 | 0.777 (0.163, 3.703) | 0.751 |  | 3 | 0.001 (4.235, 237.541) | 0.472 |  | 3 | 1.216 (0.184, 8.026) | 0.839 |
|  | Vitamin B6 | 2 | 0.962 (0.583, 1.589) | 0.881 |  |  |  |  |  |  |  |  |
|  | Vitamin C | 11 | 1.095 (0.679, 1.766) | 0.711 |  | 11 | 0.881 (0.406, 1.910) | 0.755 |  | 11 | 0.920 (0.546, 1.552) | 0.755 |
|  | Vitamin D | 6 | 1.445 (0.624, 3.346) | 0.390 |  | 6 | 1.245 (0.229, 6.758) | 0.812 |  | 6 | 1.295 (0.521, 3.215) | 0.578 |
|  | Zn | 2 | 0.802 (0.597, 1.077) | 0.142 |  |  |  |  |  |  |  |  |
| Sepsis (28 day death) | Ca | 7 | 2.618 (0.926, 7.406) | 0.070 |  | 7 | 3.868 (0.569, 26.310) | 0.225 |  | 7 | 2.871 (0.853, 9.659) | 0.088 |
|  | β-carotene | 4 | 0.781 (0.611, 0.997) | 0.047 |  | 4 | 1.165 (0.181, 7.491) | 0.887 |  | 4 | 0.771 (0.581, 1.023) | 0.072 |
|  | Fe | 3 | 1.098 (0.869, 1.387) | 0.434 |  | 3 | 1.163 (0.733, 1.844) | 0.637 |  | 3 | 1.091 (0.860, 1.384) | 0.473 |
|  | Mg | 5 | 0.882 (0.005, 170.597) | 0.963 |  | 5 | 1.157E+04 (2.028E-04, 6.597E+11) | 0.363 |  | 5 | 2.453 (0.013, 453.761) | 0.736 |
|  | Phosphorus | 3 | 0.838 (0.223, 3.153) | 0.794 |  | 3 | 25.456 (0.001, 1.127E+06) | 0.659 |  | 3 | 1.165 (0.225, 6.018) | 0.856 |
|  | Vitamin B6 | 2 | 0.789 (0.516, 1.208) | 0.276 |  |  |  |  |  |  |  |  |
|  | Vitamin C | 11 | 0.849 (0.534, 1.349) | 0.488 |  | 11 | 0.910 (0.422, 1.959) | 0.815 |  | 11 | 0.883 (0.573, 1.361) | 0.572 |
|  | Vitamin D | 6 | 0.933 (0.474, 1.838) | 0.841 |  | 6 | 1.296 (0.379, 4.429) | 0.700 |  | 6 | 1.052 (0.498, 2.222) | 0.895 |
|  | Zn | 2 | 0.921 (0.733, 1.157) | 0.480 |  |  |  |  |  |  |  |  |
| Sepsis (28 day death in critical care) | Ca | 7 | 2.265 (0.195, 26.267) | 0.513 |  | 7 | 17.505 (0.196, 1.564E+03) | 0.267 |  | 7 | 5.448 (0.312, 95.195) | 0.245 |
|  | β-carotene | 4 | 0.449 (0.253, 0.799) | 0.006 |  | 4 | 7.144 (0.089, 573.446) | 0.472 |  | 4 | 0.425 (0.211, 0.854) | 0.016 |
|  | Fe | 2 | 1.044 (0.450, 2.423) | 0.921 |  |  |  |  |  |  |  |  |
|  | Mg | 5 | 6.044E-05 (3.508E-09, 1.041) | 0.051 |  | 5 | 99.123 (2.354E-13, 4.174E+16) | 0.802 |  | 5 | 0.018 (1.091E-07, 3.051E+03) | 0.514 |
|  | Phosphorus | 3 | 0.191 (0.008, 4.344) | 0.299 |  | 3 | 108.138 (1.958E-09, 5.973E+12) | 0.774 |  | 3 | 0.185 (0.005, 7.073) | 0.364 |
|  | Vitamin B6 | 2 | 0.485 (0.177, 1.326) | 0.159 |  |  |  |  |  |  |  |  |
|  | Vitamin C | 11 | 0.860 (0.362, 2.044) | 0.733 |  | 11 | 1.122 (0.267, 4.714) | 0.878 |  | 11 | 1.142 (0.393, 3.322) | 0.807 |
|  | Vitamin D | 6 | 0.966 (0.110, 8.482) | 0.975 |  | 6 | 9.349 (0.281, 310.761) | 0.279 |  | 6 | 2.352 (0.398, 13.914) | 0.346 |

*Continued on next page*

**Supplementary Table S7. *Continued***

|  | Zn | 2 | 0.450 (0.263, 0.770) | 0.004 |  |  |  |  |  |  |  |  |
| --- | --- | --- | --- | --- | --- | --- | --- | --- | --- | --- | --- | --- |
| Streptococcal septicaemia | Ca | 6 | 0.388 (0.147, 1.021) | 0.055 |  | 6 | 0.648 (0.112, 3.758) | 0.653 |  | 6 | 0.495 (0.163, 1.504) | 0.215 |
|  | β-carotene | 4 | 1.162 (0.933, 1.447) | 0.181 |  | 4 | 0.630 (0.120, 3.314) | 0.640 |  | 4 | 1.090 (0.836, 1.422) | 0.524 |
|  | Fe | 3 | 0.865 (0.654, 1.143) | 0.308 |  | 3 | 0.803 (0.363, 1.777) | 0.684 |  | 3 | 0.820 (0.628, 1.072) | 0.147 |
|  | Mg | 4 | 0.169 (0.002, 11.518) | 0.410 |  | 4 | 0.108 (1.127E-08, 1.027E+06) | 0.811 |  | 4 | 0.238 (0.002, 32.516) | 0.567 |
|  | Phosphorus | 2 | 0.595 (0.040, 8.804) | 0.706 |  |  |  |  |  |  |  |  |
|  | Vitamin B6 | 2 | 0.892 (0.607, 1.310) | 0.561 |  |  |  |  |  |  |  |  |
|  | Vitamin C | 9 | 1.257 (0.780, 2.025) | 0.347 |  | 9 | 1.176 (0.504, 2.743) | 0.719 |  | 9 | 1.078 (0.675, 1.722) | 0.752 |
|  | Vitamin D | 5 | 1.079 (0.567, 2.052) | 0.817 |  | 5 | 1.844 (0.531, 6.405) | 0.406 |  | 5 | 1.140 (0.550, 2.364) | 0.724 |
|  | Zn | 2 | 0.986 (0.765, 1.272) | 0.915 |  |  |  |  |  |  |  |  |
| Puerperal sepsis | Ca | 6 | 0.873 (0.413, 1.846) | 0.723 |  | 6 | 1.033 (0.265, 4.024) | 0.965 |  | 6 | 0.918 (0.394, 2.135) | 0.842 |
|  | β-carotene | 4 | 0.837 (0.678, 1.033) | 0.097 |  | 4 | 1.979 (0.425, 9.202) | 0.476 |  | 4 | 0.866 (0.708, 1.059) | 0.162 |
|  | Fe | 3 | 0.900 (0.745, 1.088) | 0.277 |  | 3 | 0.735 (0.498, 1.082) | 0.363 |  | 3 | 0.868 (0.710, 1.060) | 0.164 |
|  | Mg | 4 | 20.253 (0.768, 534.043) | 0.072 |  | 4 | 1.021 (2.186E-05, 4.764E+04) | 0.997 |  | 4 | 11.822 (0.310, 450.436) | 0.184 |
|  | Phosphorus | 2 | 1.111 (0.320, 3.858) | 0.868 |  |  |  |  |  |  |  |  |
|  | Vitamin B6 | 2 | 0.891 (0.661, 1.202) | 0.450 |  |  |  |  |  |  |  |  |
|  | Vitamin C | 9 | 0.702 (0.507, 0.971) | 0.032 |  | 9 | 0.901 (0.530, 1.532) | 0.712 |  | 9 | 0.731 (0.509, 1.050) | 0.090 |
|  | Vitamin D | 5 | 1.105 (0.671, 1.819) | 0.695 |  | 5 | 1.767 (0.673, 4.642) | 0.332 |  | 5 | 1.269 (0.738, 2.181) | 0.389 |
|  | Zn | 2 | 0.894 (0.765, 1.045) | 0.158 |  |  |  |  |  |  |  |  |

The odds ratios (OR) correspond to a 1 standard deviation increase in the concentration of the micronutrient.

Abbreviations: IVW, inverse-variance weighted; Nsnp, number of SNP; OR, odds ratios; Ca, Calcium; Mg, Magnesium; Fe, iron; Zn, zinc.

**Supplementary Table S8.** Heterogeneity and pleiotropy a lysis for the causal association between circulating micronutrient and sepsis-related outcomes

| **Outcome** | **Exposure** | **Nsnp** | **Heterogeneity test** | | | | |  | **Pleiotropy test** | | | |
| --- | --- | --- | --- | --- | --- | --- | --- | --- | --- | --- | --- | --- |
|  |  |  | **IVW** | |  | **MR-Egger** | |  | **MR-Egger** | | | |
|  |  |  | **Q** | **Q_pval** |  | **Q** | **Q_pval** |  | **Intercept** | **SE** | ***P* value** | |
| Sepsis (critical care) | Ca | 7 | 3.100339 | 0.796152 |  | 2.558771 | 0.767619 |  | -0.02405 | 0.032687 | 0.494836 |  |
|  | β-carotene | 4 | 5.375208 | 0.146296 |  | 1.68657 | 0.430295 |  | -0.2945 | 0.153338 | 0.194753 |  |
|  | Fe | 3 | 5.28229 | 0.07128 |  | 3.085675 | 0.078984 |  | -0.07839 | 0.092914 | 0.553831 |  |
|  | Mg | 5 | 4.565402 | 0.471181 |  | 4.360326 | 0.359432 |  | -0.02861 | 0.065963 | 0.686847 |  |
|  | Phosphorus | 3 | 1.130238 | 0.568292 |  | 0.002133 | 0.963159 |  | 0.256392 | 0.241395 | 0.480827 |  |
|  | Vitamin B6 | 2 | 0.006409 | 0.936191 |  |  |  |  |  |  |  |  |
|  | Vitamin C | 11 | 14.56921 | 0.148575 |  | 13.79313 | 0.129877 |  | 0.019509 | 0.027415 | 0.494734 |  |
|  | Vitamin D | 6 | 5.548071 | 0.352703 |  | 5.488964 | 0.240701 |  | 0.008086 | 0.038959 | 0.845725 |  |
|  | Zn | 2 | 1.208367 | 0.271656 |  |  |  |  |  |  |  |  |
| Sepsis (28 day death) | Ca | 7 | 5.284734 | 0.507846 |  | 5.055591 | 0.409134 |  | -0.01331 | 0.027969 | 0.654113 |  |
|  | β-carotene | 4 | 0.322331 | 0.955775 |  | 0.140965 | 0.931944 |  | -0.05558 | 0.130519 | 0.711654 |  |
|  | Fe | 3 | 0.276636 | 0.870822 |  | 0.19669 | 0.657406 |  | -0.01268 | 0.044844 | 0.824576 |  |
|  | Mg | 5 | 8.072375 | 0.152292 |  | 6.23114 | 0.182539 |  | -0.07294 | 0.067089 | 0.338084 |  |
|  | Phosphorus | 3 | 1.448657 | 0.48465 |  | 1.036689 | 0.308593 |  | -0.13153 | 0.208654 | 0.641924 |  |
|  | Vitamin B6 | 2 | 0.008674 | 0.925798 |  |  |  |  |  |  |  |  |
|  | Vitamin C | 11 | 18.9663 | 0.040693 |  | 18.85689 | 0.026438 |  | -0.00622 | 0.027225 | 0.82436 |  |
|  | Vitamin D | 6 | 2.584091 | 0.763781 |  | 2.188991 | 0.701045 |  | -0.01779 | 0.028308 | 0.563737 |  |
|  | Zn | 2 | 0.025983 | 0.871942 |  |  |  |  |  |  |  |  |
| Sepsis (28 day death in critical care) | Ca | 7 | 2.402237 | 0.879244 |  | 1.26889 | 0.9381 |  | -0.06978 | 0.065548 | 0.335749 |  |
|  | β-carotene | 4 | 1.932488 | 0.586537 |  | 0.376911 | 0.828237 |  | -0.38364 | 0.307597 | 0.33856 |  |
|  | Fe | 2 | 2.20518 | 0.137548 |  |  |  |  |  |  |  |  |
|  | Mg | 5 | 4.402868 | 0.492984 |  | 3.645558 | 0.456094 |  | -0.11012 | 0.126535 | 0.43328 |  |
|  | Phosphorus | 3 | 0.361514 | 0.834638 |  | 0.105118 | 0.745772 |  | -0.24439 | 0.48265 | 0.701604 |  |
|  | Vitamin B6 | 2 | 0.000954 | 0.975353 |  |  |  |  |  |  |  |  |

*Continued on next page*

**Supplementary Table S8. *Continued***

|  | Vitamin C | 11 | 11.64657 | 0.309406 |  | 11.3717 | 0.251089 |  | -0.02345 | 0.050275 | 0.652 |
| --- | --- | --- | --- | --- | --- | --- | --- | --- | --- | --- | --- |
|  | Vitamin D | 6 | 9.289451 | 0.098061 |  | 5.880726 | 0.208235 |  | -0.12287 | 0.080695 | 0.2025 |
|  | Zn | 2 | 0.911225 | 0.33979 |  |  |  |  |  |  |  |
| Streptococcal septicaemia | Ca | 6 | 1.885935 | 0.864692 |  | 1.417905 | 0.841077 |  | -0.01848 | 0.027012 | 0.531473 |
|  | β-carotene | 4 | 1.069138 | 0.784529 |  | 0.538791 | 0.763841 |  | 0.084776 | 0.116411 | 0.542185 |
|  | Fe | 3 | 2.647902 | 0.266082 |  | 2.535793 | 0.111291 |  | 0.014654 | 0.069695 | 0.868064 |
|  | Mg | 4 | 2.694801 | 0.441112 |  | 2.690241 | 0.260508 |  | 0.003657 | 0.062818 | 0.958868 |
|  | Phosphorus | 2 | 2.830983 | 0.092462 |  |  |  |  |  |  |  |
|  | Vitamin B6 | 2 | 0.898661 | 0.343141 |  |  |  |  |  |  |  |
|  | Vitamin C | 9 | 13.94016 | 0.083339 |  | 13.86618 | 0.053613 |  | 0.005171 | 0.026757 | 0.852247 |
|  | Vitamin D | 5 | 2.701459 | 0.608959 |  | 1.730147 | 0.630252 |  | -0.02757 | 0.027971 | 0.39702 |
|  | Zn | 2 | 1.613254 | 0.204035 |  |  |  |  |  |  |  |
| Puerperal sepsis | Ca | 6 | 0.733796 | 0.981062 |  | 0.650166 | 0.957329 |  | -0.00606 | 0.020946 | 0.786805 |
|  | β-carotene | 4 | 4.601344 | 0.203427 |  | 2.853039 | 0.240143 |  | -0.11938 | 0.107831 | 0.383595 |
|  | Fe | 3 | 2.020713 | 0.364089 |  | 0.635425 | 0.425372 |  | 0.040046 | 0.034025 | 0.448358 |
|  | Mg | 4 | 1.827193 | 0.609035 |  | 1.500181 | 0.472324 |  | 0.02404 | 0.042038 | 0.625128 |
|  | Phosphorus | 2 | 0.000352 | 0.985038 |  |  |  |  |  |  |  |
|  | Vitamin B6 | 2 | 0.541947 | 0.461627 |  |  |  |  |  |  |  |
|  | Vitamin C | 9 | 10.58039 | 0.226627 |  | 8.883737 | 0.261111 |  | -0.0193 | 0.016693 | 0.285516 |
|  | Vitamin D | 5 | 3.051734 | 0.549205 |  | 1.81354 | 0.611993 |  | -0.02415 | 0.021704 | 0.346959 |
|  | Zn | 2 | 0.704587 | 0.401247 |  |  |  |  |  |  |  |

Abbreviations: IVW, inverse-variance weighted; Nsnp, number of SNP; Ca, Calcium; Mg, Magnesium; Fe, iron; Zn, zinc.

**Supplementary Table S9.** MR-PRESSO and Steiger direction test for the causal association between circulating micronutrient and sepsis-related outcomes

| **Exposure** | **Outcome** | **MR-PRESSO pval** | **Correct_causal_direction** | **Steiger_pval** |
| --- | --- | --- | --- | --- |
| Fe | Sepsis (UK Biobank) | NA | TRUE | 7.8659E-146 |
| β-carotene | Sepsis (28 day death in critical care) | 0.958 | TRUE | 1.14195E-66 |
| β-carotene | Sepsis (28 day death) | 0.64 | TRUE | 1.82582E-67 |
| Vitamin C | Puerperal sepsis | 0.216 | TRUE | 6.5313E-124 |
| Zn | Sepsis (28 day death in critical care) | NA | TRUE | 4.24156E-28 |

Abbreviations: Fe, iron; Zn, zinc.

**Supplementary Table S10.** The post hoc mendelian randomization analyses of zinc as protective factor on the risk of Sepsis (28 day death in critical care)

| **Exposure** | **Outcome** | **Method** | **Nsnp** | **P value** | **OR (95% CI)** | **Heterogeneity test** | **Pleiotropy test** |
| --- | --- | --- | --- | --- | --- | --- | --- |
|  |  |  |  |  |  | **Cochran’s Q** | **egger_intercept_pval** |
| Zn | Sepsis (28 day death in critical care) | MR Egger | 3 | 0.639 | 0.546 (0.085, 3.524) | 0.265 | 0.915 |
|  |  | Weighted median | 3 | 0.0265 | 0.521 (0.293, 0.927) |  |  |
|  |  | IVW | 3 | 0.00316 | 0.483 (0.298, 0.783) | 0.531 |  |
|  |  | Simple mode | 3 | 0.31869 | 0.598 (0.279, 1.285) |  |  |
|  |  | Weighted mode | 3 | 0.2526 | 0.574 (0.289, 1.138) |  |  |

SNPs with r^2^ < 0.001 within 10,000 kb windows and *P* < 5E-07 were used as genetic instruments for the exposure.

Abbreviations: Zn, Zinc; CI, confidence interval; IVW, inverse-variance weighted; Nsnp, number of SNP; OR, odds ratios.

**Supplementary Table S11.** Phenome-wide association analysis of genetic instruments for zinc.

| **SNP** | **A1** | **A2** | **Trait** | **Efo** | **Study** | **PMID** | **Ancestry** | **Year** | **Beta** | **StdErr** | ***P* value** | **Direction** | **N** |
| --- | --- | --- | --- | --- | --- | --- | --- | --- | --- | --- | --- | --- | --- |
| rs1532423 | G | A | High light scatter percentage of red cells | EFO_0004586 | Astle W | 27863252 | European | 2016 | 0.02297 | 0.003614 | 2.06E-10 | + | 173480 |
|  |  |  | High light scatter reticulocyte count | EFO_0004586 | Astle W | 27863252 | European | 2016 | 0.02363 | 0.003614 | 6.27E-11 | + | 173480 |
|  |  |  | Mean corpuscular hemoglobin concentration | EFO_0004586 | Astle W | 27863252 | European | 2016 | 0.01957 | 0.00349 | 2.05E-08 | + | 173480 |
|  |  |  | Reticulocyte count | EFO_0004586 | Astle W | 27863252 | European | 2016 | 0.02292 | 0.00362 | 2.43E-10 | + | 173480 |
|  |  |  | Reticulocyte fraction of red cells | EFO_0004586 | Astle W | 27863252 | European | 2016 | 0.02213 | 0.003618 | 9.53E-10 | + | 173480 |
|  |  |  | Zinc levels | - | Evans DM | 23720494 | European | 2013 |  |  | 6.40E-12 |  | 2603 |
|  |  |  | Blood trace element Zn levels | EFO_0005268 | Evans DM | 23720494 | European | 2013 | -0.178 | 0.02587 | 6.00E-12 | - | - |
|  |  |  | Reticulocyte fraction of red cells | EFO_0007986 | Astle W | 27863252 | European | 2016 | 0.02213 | 0.003622 | 1.00E-09 | + | - |
| rs2120019 | C | T | Mean corpuscular volume | EFO_0004586 | Astle W | 27863252 | European | 2016 | -0.02818 | 0.004473 | 2.99E-10 | - | 173480 |
|  |  |  | Zinc levels | - | Evans DM | 23720494 | European | 2013 |  |  | 1.55E-18 |  | 2603 |
|  |  |  | Blood trace element Zn levels | EFO_0005268 | Evans DM | 23720494 | European | 2013 | 0.287 | 0.03277 | 2.00E-18 | + | - |

Only diseases and traits with <5E-08 and r^2^ > 0.8 are reported

Abbreviations: A1, the effect allele (aligned to the + strand) for the input SNP; A2, the non-effect allele (aligned to the + strand) for the input SNP; Beta, association between the trait and the SNP expressed per additional copy of the effect allele (odds ratios are given on the log-scale); Efo, the EFO ontology term for the phenotype or disease; PMID, PubMed ID; StdErr, standard error; Study, the name of the consortium/lead author of the study; Trait, phenotype or disease; Year, the year the study was published.

**Supplementary Table S12.** Reverse associations for Sepsis (28 day death in critical care) on Zn

| **Exposure** | **Outcome** | **Nsnp** | **Beta** | **IVW analysis** | |
| --- | --- | --- | --- | --- | --- |
|  |  |  |  | **95% CI** | ***P*** |
| Sepsis (28 day death in critical care) | Zn | 2 | -0.0142 | (-0.115, 0.087) | 0.783 |

Abbreviations: CI, confidence interval; IVW, inverse-variance weighted; Nsnp, number of SNP.
